# Kinetically and thermodynamically controlled one-pot growth of gold nanoshells with NIR-II absorption for multimodal imaging-guided photothermal therapy

**DOI:** 10.1186/s12951-023-01907-1

**Published:** 2023-04-28

**Authors:** Ming Chen, Xiao-Tong Chen, Lian-Ying Zhang, Wei Meng, Yong-Jian Chen, Ying-Shan Zhang, Zhi-Cong Chen, Hui-Min Wang, Chun-Mei Luo, Xiu-Dong Shi, Wen-Hua Zhang, Mao-Sheng Wang, Jin-Xiang Chen

**Affiliations:** 1 The People’s Hospital of Gaozhou, Maoming, 525200 China; 2 Guangdong Provincial Key Laboratory of New Drug Screening, School of Pharmaceutical Sciences, Southern Medical University, Guangzhou, 510515 China; 3 College of Chemistry, Chemical Engineering and Materials Science, Soochow University, Suzhou, 215123 China

**Keywords:** Controllable growth, Gold nanoshells, NIR-II materials, Multimodal imaging, Photothermal therapy

## Abstract

Since the successful clinical trial of AuroShell for photothermal therapy, there is currently intense interest in developing gold-based core-shell structures with near-infrared (NIR) absorption ranging from NIR-I (650–900 nm) to NIR-II (900–1700 nm). Here, we propose a seed-mediated successive growth approach to produce gold nanoshells on the surface of the nanoscale metal–organic framework (NMOF) of UiO-66-NH_2_ (UiO = the University of Oslo) in one pot. The key to this strategy is to modulate the proportion of the formaldehyde (reductant) and its regulator / oxidative product of formic acid to harness the particle nucleation and growth rate within the same system. The gold nanoshells propagate through a well-oriented and controllable diffusion growth pattern (points → facets → octahedron), which has not been identified. Most strikingly, the gold nanoshells prepared hereby exhibit an exceedingly broad and strong absorption in NIR-II with a peak beyond 1300 nm and outstanding photothermal conversion efficiency of 74.0%. Owing to such superior performance, these gold nanoshells show promising outcomes in photoacoustic (PA), computed tomography (CT), and photothermal imaging-guided photothermal therapy (PTT) for breast cancer, as demonstrated both in vitro and in vivo.

**Supplementary Information:**

The online version contains supplementary material available at 10.1186/s12951-023-01907-1.

## Introduction

Since AuroShell (gold nanoparticles deposit on silica) was approved for clinical trials as a photothermal destruction agent for solid tumors [1–5], extensive efforts have been made to use different templates to grow gold nanoshells with diverse morphologies to obtain near-infrared (NIR) photothermal therapeutic agent [6–8]. Among the various studies, the cladding of gold nanoshells onto the surface of the silica core was relatively mature [9–11]. The formation of such core-shell structures generally involves three sequential steps: (i) silica core surface modified with -NH_2_, (ii) a small number of gold ions bound to the surface were reduced to form gold seeds with uniform and suitable sizes, and (iii) the gold seeds act as nucleation sites to initiate the growth of gold nanoshells by careful modulation of the reaction conditions. The ultraviolet-visible (UV-Vis) absorption bands of these gold seeds are within the visible range, whereas those of the gold nanoshells fall in the NIR region. The NIR absorption capability of these hybrid materials is considered critical for biological applications as living cells and tissues have low light scattering and adsorption in this region, which is ideal for photothermal therapy (PTT) [11].

Recently, gold nanoshells have been explored to grow onto the surface of nanoscale metal–organic frameworks (NMOFs). Compared to silica, NMOF is a superior type of template with abundant surface morphologies [12] and controllable particle sizes [13], as well as high tolerance toward further surface functionalization [14], making them attractive candidates for the coating of gold nanoshells [15, 16]. However, current protocols for growing gold nanoshells on NMOF and other templates such as silica [9–11], silver nanoparticles [17], MnFe_2_O_4_ nanoparticles [18], and superparamagnetic iron oxide nanoparticles [19], usually result in the random ornament of gold seeds and thus uncontrollable growth of gold shells as a result. With the unfavorable thickness and uniformity, the constructed gold nanoshells usually have an absorption peak located in the NIR-I window (650–900 nm) [20–23]. Compared with NIR-I, materials that exhibit absorptions in the NIR-II region (900–1700 nm) are highly desirable as they could yield improved photon penetration depth [24], reduced background autofluorescence [25–27], lower photon absorption and scattering [28], and higher maximum permissible exposure [29] for more effective PTT and photoacoustic imaging (PAI) for cancers. Therefore, it remains a formidable challenge to obtain gold nanoshells with a NIR-II absorption window [30, 31].

**Scheme 1:**
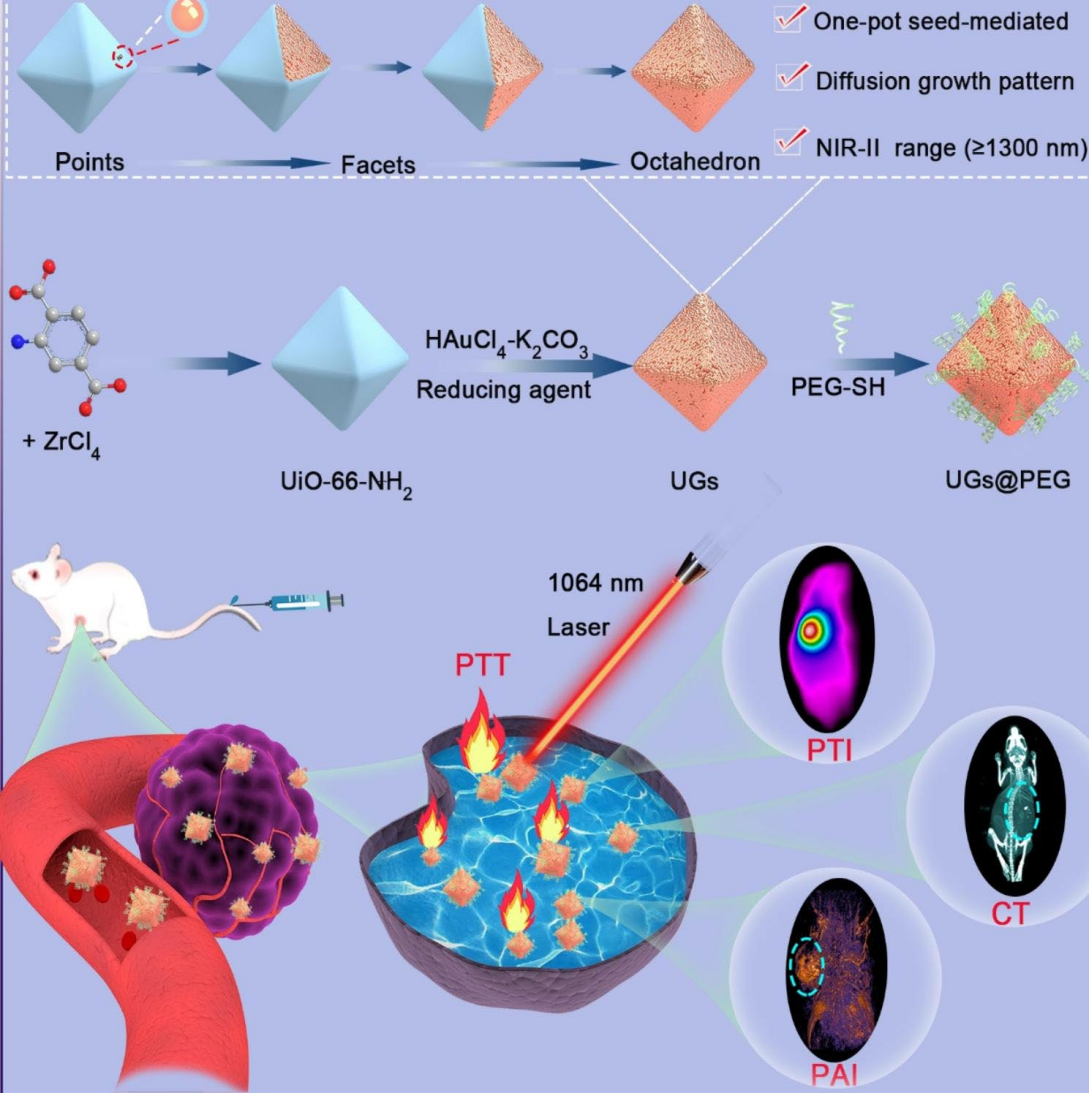
Schematic presentation of the preparation process, photothermal imaging (PTI), photoacoustic imaging (PAI), computed tomography (CT), and imaging-guided NIR-II photothermal therapy (PTT) of UGs

In this work, we propose a kinetically and thermodynamically controlled [32–35] one-pot seed-mediated growth approach to coat gold nanoparticles on NMOF of UiO-66-NH_2_ to obtain island-on-core, core-shell, and island-on-shell gold nanostructures in the Volmer–Weber (3D island) mode, Frank-van der Merwe (layer-by-layer) mode, and Stranski-Krastanov (mixed) mode, respectively [36, 37]. The gold nanoshells on UiO-66-NH_2_ (denoted as UGs) formed through a well-oriented and controllable diffusion growth pattern (points → facets → octahedron) (Scheme 1). Critically, the gold nanoshells exhibit an exceedingly long, broad, and strong absorption in NIR-II with a peak beyond 1300 nm and outstanding photothermal conversion efficiency of 74.0%. The superior optical properties of UGs allow us to further investigate the performance of gold nanoshells in photoacoustic (PA), computed tomography (CT), and photothermal imaging-guided PTT for tumors in vitro and in vivo. This controlled growth of gold nanoparticles demonstrated herein provides the opportunity to merge the sequential two-step seed-mediated growth into a one-pot cascade synthesis and presents a brand-new avenue toward the preparation of highly promising gold nanoshells for multi-modal cancer theranostics and beyond.

## Methods

### Materials and measurements

2-Aminoterephthalic acid (NH_2_-BDC, 99%), ZrCl_4_, polyvinyl pyrrolidone (PVP, Mw = 40000), K_2_CO_3_, PEG-SH (Mw = 4000), acetic acid, formaldehyde, and formic acid were purchased from Aladdin (Shanghai, China). Fetal bovine serum (FBS), penicillin-streptomycin liquid, PBS, and 3-(4,5-dimethyl-2-thiazolyl)-2,5-diphenyl-2- Htetrazolium bromide (MTT) were purchased from Procell (Wuhan, China). Chloroauric acid (HAuCl_4_, 99.9+%) was purchased from Adamas. N, N’-Dimethylformamide (DMF), and ethanol were purchased from Shenzhen Cool Thai technology co.LTD. Calcein AM/PI and Annexin V-FITC Apoptosis Detection Kit were purchased from Beyotime Biotechnology (Shanghai, China). Unless otherwise stated, all reagents and solvents were commercially available and used without further purification.

UV-Vis absorption spectra were captured by a Perkin Elmer LAMBDA 25 s.pectrometer. Synthesis of UiO-66-NH_2_ was conducted in an autoclave (Memmert UN 30 plus). The particle size distribution and zeta potential were measured by dynamic light scatting analysis (DLS, Malvern Nano ZS90, UK). Water was purified with an ELGA Lab Water purifier (18.2 MΩ cm, PURELAB). TomoWave 3D optoacoustic Imaging System was employed for in vivo PA imaging. All of the PA images were obtained with a 1064 nm laser illumination. CT images were obtained by micro PET/CT (Siemens inveon, Germany). Transmission electron microscopy (TEM, JEOL JEM-2100 F, 100 kV) and scanning electron microscopy (SEM, SU8020 EMAXevolutin X-Max80 from Zeiss, Japan) were performed to observe the morphology of the materials. Cellular fluorescence imaging was observed with an Olympus FV1000 confocal laser scanning microscope (CLSM) imaging system (Zeiss, Japan). Flow cytometry analysis was conducted with BD Beckman Coulter flow cytometer (Brea, CA) and FlowJo Software (TreeStar, Ashland, OR). The crystalline structures of NPs were recorded by a powder X-ray diffractometer (PXRD, Bruker, Germany) with laser (VCL-1064nmM0-2). Time-Domain (FDTD) simulations were carried out using FDTD Solutions software (Lumerical Co.Ltd), with the plane wave at different wavelengths used as the light source. The periodic boundary was used along the *x*-axis and *y*-axis, and the perfect match layer (PML) boundary was used along the *z*-axis. Mesh size was set as 1 nm for the structure during FDTD simulations.

### Synthesis of UiO-66-NH_2_

Specifically, the solution of ZrCl_4_ (42 mg, 0.18 mM) in DMF (2.5 mL) was added into the solution of NH_2_-BDC (33 mg, 0.18 mM) in DMF (1.0 mL) followed by an additional 0.1 mL of deionized (DI) water. Then, acetic acid (1.7 mL) was introduced and the mixture was sonicated for 30 min. The final suspension was transferred into an autoclave for crystallization at 120 °C for 2 h. After smooth cooling to room temperature, the precipitate was filtered and washed with DMF and ethanol three times by centrifugation at 8260 rpm and re-dispersed in 30 mL ethyl alcohol for further use.

### Synthesis of UiO-66-NH_2_@Gold-shells (UGs)

#### Controlling the reaction kinetics

The aged HAuCl_4_ was prepared using the previously reported method with optimization [15]. Briefly, aqueous solutions of K_2_CO_3_ (7 mL, 1.13 mg mL^− 1^) and HAuCl_4_ (0.30 mL, 10 mg mL^− 1^) were mixed under vigorous stirring for 15 min in the dark. Then, PVP (0.3 mL, 1.0 mg mL^− 1^) and the as-prepared UiO-66-NH_2_ (0.32 mL, 1.5 mg mL^− 1^) were mixed and further with 70 µL regulator (formic acid/formaldehyde, (*v*/*v*)% = 0%, 0.02%, 0.04%, 0.06%, 0.08%, 0.1%). The formed mixture was quickly introduced into the aged HAuCl_4_ solution and stirred vigorously to initiate the growth of UGs. The whole solution was kept at room temperature for about 30 min. During the reaction process, the color of the solution changed from light pink at the beginning to gray and finally to oxford-blue, suggesting that the gold shells were coated on the surface of UiO-66-NH_2_. Ultimately, the optimum volume ratio of the reaction-reducing agent (formic acid/formaldehyde) was determined by UV-Vis spectroscopy.

#### Optimizing the experimental conditions

With the best volume ratio between formic acid and formaldehyde (70 µL, *v*/*v* = 0.06%) and the fixed amount of UiO-66-NH_2_ (0.32 mL, 1.5 mg mL^− 1^), we investigated the effect of different amounts of HAuCl_4_ (10 mg mL^− 1^) on the formation of gold-shells with the volume of 0.10, 0.15, 0.20 0.25, 0.30, 0.35, 0.40, 0.45, and 0.50 mL. The UV-vis spectra were used to determine the best amount of HAuCl_4_.

With the best volume ratio between formic acid and formaldehyde (70 µL, *v*/*v* = 0.06%) and the fixed amount of UiO-66-NH_2_ (0.32 mL, 1.5 mg mL^− 1^) and the above-explored amount of HAuCl_4_ (0.30 mL, 10 mg mL^− 1^), we further investigated the effect of different amount of K_2_CO_3_ (1.13 mg mL^− 1^) with 3, 5, 7, 9, 11, and 13 mL on the formation of gold-shells. The UV-vis spectra were used to determine the best amount of K_2_CO_3_.

With the best volume ratio between formic acid and formaldehyde (70 µL, *v*/*v* = 0.06%) and the fixed amount of UiO-66-NH_2_ (0.32 mL, 1.5 mg mL^− 1^), HAuCl_4_ (0.30 mL, 10 mg mL^− 1^) and K_2_CO_3_ (7 mL, 1.13 mg mL^− 1^), we explored the effect of different PVP (1 mg mL^− 1^) dosages (0, 0.025, 0.05, 0.1, 0.3, 0.5, and 1.0 mL) on gold-shells synthesis. The UV-vis spectra were used to determine the best amount of PVP.

### Synthesis of UGs@PEG

PEG-SH (1 mL, 10 mg mL^− 1^) was added to a beaker containing 3 mL of UGs (1.5 mg mL^− 1^) solution under the most optimal conditions. UGs@PEG was obtained by stirring for 24 h at room temperature. After that, the product was washed with deionized water three times by centrifugation at 8500 rpm and re-dispersed in 6 mL of deionized water for further use.

### Photothermal properties of UGs@PEG in vitro

UGs@PEG dispersed in deionized water at different concentrations (0, 50, 100, 150, and 200 µg mL^− 1^) was exposed to 1064 nm laser irradiation with a power intensity of 1.0 W cm^− 2^. Then 1.5 mL of deionized water was used as the control group. The infrared camera (FLIR TM A325SC camera) was used to measure and record the temperature and thermal images every 20 s. In addition, UGs@PEG solution (200 µg mL^− 1^) was exposed to 1064 nm laser irradiation with different power intensities (0.5, 1.0, 1.5, and 2.0 W cm^− 2^) and the temperature changes at different times were recorded simultaneously.

The photothermal conversion efficiency of UGs@PEG was calculated as follows:

$$\eta {\rm{ = }}\frac{{{\rm{hS (}}{{\rm{T}}_{{\rm{max}}}}{\rm{ - }}{{\rm{T}}_{{\rm{surr}}}}{\rm{) - }}{{\rm{Q}}_{{\rm{dis}}}}}}{{{\rm{I (1 - }}{{10}^{ - {A_{1064}}}}{\rm{)}}}}$$

$${\rm{hS = }}\frac{{{{\rm{m}}_{\rm{D}}}{{\rm{c}}_{\rm{D}}}}}{{{\tau _{\rm{s}}}}}$$

$$\theta {\rm{ = }}\frac{{{\rm{T - }}{{\rm{T}}_{{\rm{surr}}}}}}{{{{\rm{T}}_{{\rm{max}}}}{\rm{ - }}{{\rm{T}}_{{\rm{surr}}}}}}$$

$$t = - {\tau _s}In(\theta )$$

T_max_ and T_surr_ represent the maximum temperature and the ambient temperature respectively of the surroundings. Q_dis_ is the heat loss caused by the absorption of light by solvent and container, and its value is about 0 mW. I (W) is the laser power density, in the unit of W cm^− 2^. A_1064_ is the absorbance value of the sample at 1064 nm, without unit. h is the heat transfer coefficient and the unit is Wcm^2^·K^− 1^. S is the surface area of the container and the unit is m^2^. τ_s_ stands for the time constant. m_D_ is the solvent mass and its value is 1 g. c_D_ represents the specific heat capacity of water and its value is 4.2 J g^− 1^. The laser with a wavelength of 1064 nm and a safety power of 1.0 W cm^− 2^ was used to irradiate the temperature for 5 min, and then the temperature was decreased for 10 min.

### Cell culture and cytotoxicity assay

Human mammary epithelial cells (MCF-10 A) and mouse fibroblasts (L929) were cultured in DMEM (Gibico, USA) culture medium supplemented with 10% FBS, 1% penicillin-streptomycin at 37ºC in 5% CO_2_. Mouse breast cancer cells (4T1) were cultured in RPMI 1640 media (Procell) supplemented with 10% FBS, and 1% penicillin-streptomycin at 37ºC in 5% CO_2_.

MCF-10 A cells, L929 cells, or 4T1 cells were seeded in 96-well plates with a density of 0.5 × 10^4^ cells per well respectively. After 24 h incubation, the fresh medium containing different concentrations of UGs@PEG was added to MCF-10 A cells and L929 cells and further incubated for 24 h. The PBS + NIR and UGs@PEG + NIR group of 4T1 cells were further exposed to laser irradiation (1064 nm, 1.5 W cm^− 2^, 3 min) then for 8 h incubation. Finally, the cell viability was evaluated by the standard MTT assay.

### Cell apoptosis assay

To explore the type of cell death induced by diverse treatments as mentioned above, an apoptosis detection assay was carried out. Briefly, 4T1 cells with PBS, PBS + NIR, UGs@PEG, and UGs@PEG + NIR treatments were collected, treated with an annexin V-FITC/PI apoptosis detection kit, and then quantitated using a flow cytometer.

### Live-dead staining in vitro

4T1 cells were seeded in 6-well plates with a density of 3.0 × 10^5^ cells per well and cultured for 24 h. Then the cells were treated with different groups (PBS, PBS + NIR, 64 µg mL^− 1^ UGs@PEG, and 64 µg mL^− 1^ UGs@PEG + NIR). Upon incubation for a further 8 h, the 4T1 cells (PBS + NIR and UGs@PEG + NIR) were irradiated under a 1064 nm NIR laser for 3 min (1.5 W cm^− 2^) and further cultured for 16 h. Afterward, the cells of each group were incubated with Calcein-AM (Ex 494 nm, Em 517 nm) and PI (Ex 535 nm, Em 617 nm) for 20 min, and the cells were washed three times with PBS (pH 7.4, 10 mM) and then observed using CLSM.

### Animal model

All the female Balb/c nude mice were provided by Southern Medical University. All animal experiment procedures were approved by the guidelines from the Animal Care Ethics Commission of Southern Medical University (Permit Number: 44,002,100,031,030). To establish a 4T1 tumor model, the mice were hypodermically injected with a suspension of 4T1 cells (2 × 10^6^) in PBS (100 µL).

### In vitro and in vivo photoacoustic (PA) imaging

For in vitro PA imaging, UGs@PEG dissolved in deionized water with different concentrations (0, 32, 63, 125, 250, and 500 µg mL^− 1^) were introduced into a facility for PA signals using the PA equipment (LOIS 3D TomoWave Laboratories, USA). Then we also tested various concentrations (0, 32, 63, 125, 250, and 500 µg mL^− 1^) in the front and cross-section of photoacoustic tubes under a 1064 nm laser. For in vivo PA imaging, UGs@PEG dissolved in PBS (100 µL, 6 µg mL^− 1^) was intratumorally injected into the 4T1 breast tumor-bearing mice. Then, the time-dependent PA imaging on tumor-bearing mice was performed at different time intervals (0 h, 3 h, 6 h, 9 h, 12 h, 24 h, and 36 h).

For biodistribution studies, the tumor-bearing mice were injected with Te–Gd (100 *µ*L, 10 mg Te kg^− 1^) through the tail vein. Then, the mice were euthanized for analysis of Te–Gd distribution in the major organs and tumors by the ICP-MS method.

### In vitro and in vivo computed tomography (CT) imaging

UGs@PEG dissolved in deionized water and iohexol solutions with the same concentrations (UGs@PEG or iohexol: 0, 1, 2, 4, 8, and 16 mg mL^− 1^) were used for in vitro CT imaging. For in vivo CT imaging, UGs@PEG dissolved in PBS (100 *µ*L, 6 mg mL^− 1^) was intratumorally or intravenously injected into the 4T1 tumor-bearing mice. Then, the CT imaging on tumor-bearing mice was performed at different time intervals (0 h, and 24 h) using the Siemens inveno micro PET/CT operated at 80 kV and 50 µA. The tumor-bearing mice were injected with UGs@PEG (20 mg kg^− 1^) through the tail vein. Then, the mice were euthanized for analysis of the Au distribution of the major organs and tumors by the ICP-MS method.

### Hemolysis assay and histology analysis

To evaluate the biocompatibility of nanomaterials in animals, we performed hemolysis tests. The blood samples were collected from the healthy mice of eyeballs. Subsequently, a 1 mL blood sample was diluted with 10 mL of PBS, and the red blood cells (RBCs) were separated from the serum by centrifugation (2500 rpm, 30 min). After washing with PBS at least four times, the RBCs (10 *µ*L) were diluted with 1 mL of PBS. Then the RBCs were incubated with different concentrations of UGs@PEG solution (0, 5, 10, 20, 50, 100, 200, and 300 µg mL^− 1^) at 37ºC for 2 h and placed on a shaker, and the PBS and 1% Triton X-100 were used as negative and positive controls. After 2 h, the mixtures were centrifuged at 3000 rpm for 10 min. Finally, the absorbances of supernatants at 570 nm were recorded by a microplate reader to calculate the percent hemolysis.

### In vivo antitumor studies

When the tumor volume reached around 100 mm^3^, the mice were randomly divided into four groups (n = 5): PBS, PBS + NIR, UGs@PEG, and UGs@PEG + NIR and i.v. injected with different formulations. The injection dose was 30 mg kg^− 1^. Groups PBS + NIR and UGs@PEG + NIR were irradiated by 1064 nm laser (1 W cm^− 2^, 10 min) at 24 h and the infrared camera was used to measure and record the temperature every 30 s and thermal images every 2 min. Besides, tumor volume and body weight of mice were recorded every 2 days for 15 days. Tumor volumes (V) were calculated by using the equation of width^2^ × length/2. After different treatments, their major organs (heart, liver, spleen, lung, and kidney) were excised for hematoxylin and eosin (H&E) staining. The tumor tissues were collected for hematoxylin and eosin (H&E), Ki-67, and TUNEL staining.

### Statistical analysis

The test data were performed to calculate the mean value and standard deviation (mean ± SD). The statistical analysis of different groups was compared by the Student’s t-test. p < 0.05 (*), p < 0.01 (**), and p < 0.001 (***) were regarded as statistically significant.

## Results and discussion

Based on theoretical analysis of the seed-mediated growth [36–39], the growth pattern is determined by the ratio between the rate of deposition (*V*_dep_) and the rate of surface diffusion (*V*_diff_). In this work, the rate of deposition (*V*_dep_) is mainly modulated by the strength of the reductant and reaction temperature, and *V*_diff_ is dependent on the reaction temperature. In the first set of experiments, we chose formic acid as the formaldehyde activity regulator. Formaldehyde can be oxidized to formic acid, which slows down the reduction rate of Au^3+^ to Au nanoparticles. In addition, the level of formic acid can regulate the pH value of the reaction system and further modulate the formation rate of Au nanoparticles. We precisely regulated the reduction rate by adjusting the volume ratio of formic acid to formaldehyde (0%, 0.02%, 0.04%, 0.06%, 0.08%, 0.1%).

**Fig. 1:**
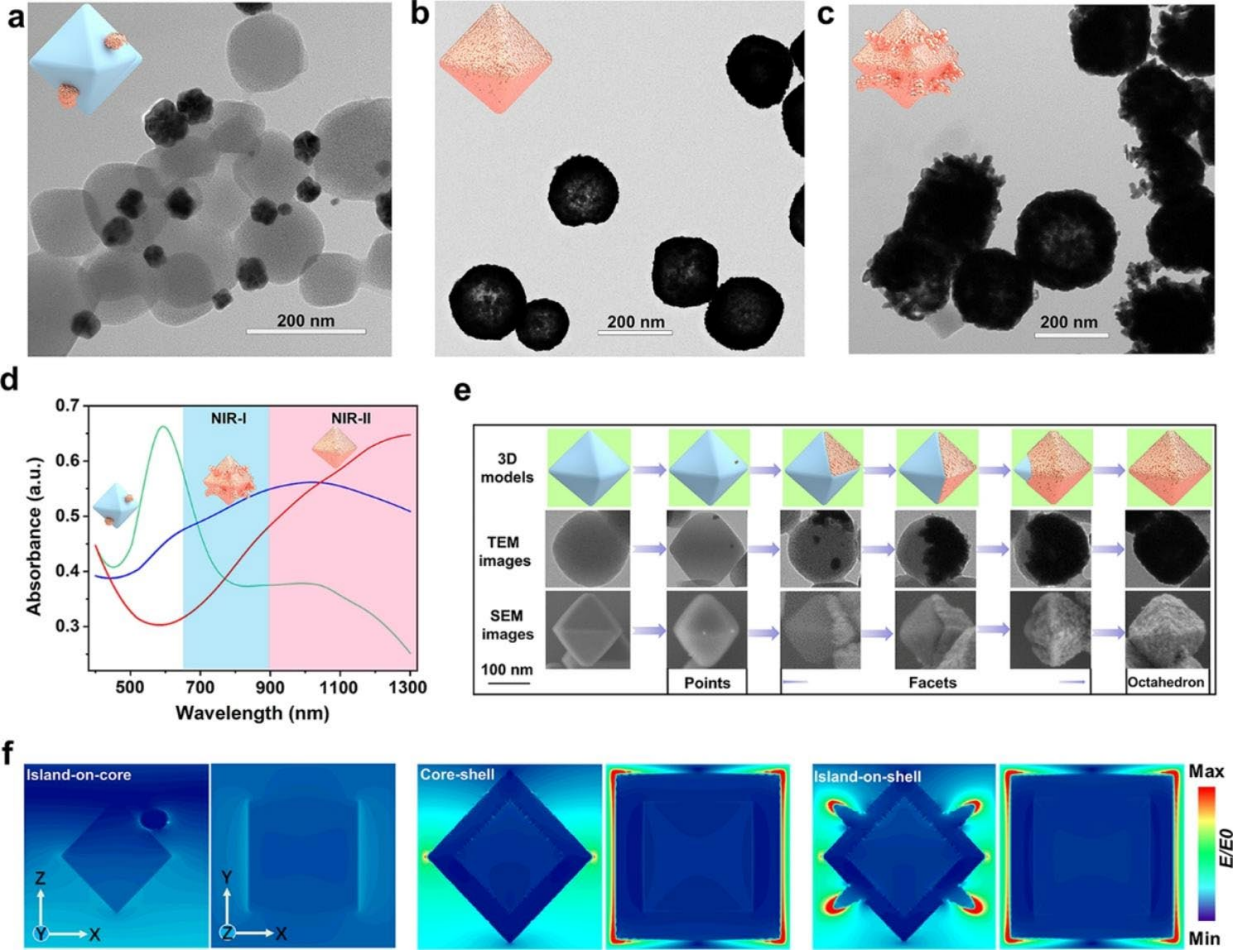
(a) The TEM images of island-on-core nanoparticles without formic acid (0%) at 25 °C. (b) The TEM images of core-shell nanoparticles with the ratio of formic acid to formaldehyde being 0.06% at 25 °C. (c) The TEM images of island-on-shell nanoparticles with the ratio of formic acid to formaldehyde being 0.06% at 75 °C. (d) The UV-Vis spectra of island-on-core (green line), core-shell (red line), and island-on-shell nanoparticles (blue line). (e) The schematic representation (3D models), TEM, and SEM images of different stages involved in the growth of UGs. (f) Electric field distributions of the island-on-core, core-shell, and island-on-shell nanostructures with an incidence of 1064 nm laser at the *y* and *z*-direction

As indicated by transmission electron microscopy (TEM) in Fig. 1a, when the reaction is carried out at 25 °C without the presence of formic acid, the rate of gold nanoparticle formation featured *V*_dep_ > *V*_diff_ and the particles randomly deposited on the surface of UiO-66-NH_2_ to form island-on-core nanostructures in the Volmer–Weber (island) mode. With the volume ratio of formic acid adjusted to the most appropriate ratio of 0.06%, the formation rate of gold nanoparticles showed *V*_dep_ < *V*_diff_ to give gold core-shell nanostructures in the Frank–van der Merwe (layer-by-layer) mode instead of the Volmer–Weber (island) mode (Fig. 1b).

In the second set of experiments, we varied the reaction temperature (0, 25, 50, and 75 °C) while keeping formic acid the same as the optimized synthesis condition (0.06%) to modulate the rates of atom deposition and surface diffusion (*V*_dep_/*V*_diff_). At a relatively low temperature of 0 or 25 °C, gold nanoparticles were formed on the surface of UiO-66-NH_2_ to form core-shell nanostructures, and more such structures were obtained at 25 °C (Fig. 1b) than at 0 °C (Figure S1a). However, when the reaction temperature was raised to 50 °C, gold nanoparticles gradually form island-on-shell nanostructures in Stranski-Krastanov mode (mixed mode) (Figure S1b), and the formation of island-on-shell nanostructures further dominate at a higher reaction temperature of 75 °C (Fig. 1c).

The effect of the formic acid and the reaction temperature on the final formation of gold nanoparticles was characterized by the UV-Vis spectra. When the gold nanoparticles were randomly deposited on the surface of UiO-66-NH_2_ to form island-on-core nanostructures, the UV-Vis spectrum shows a peak at 600 nm (Fig. 1d, green line). The gold nanoshells formed on the octahedral surface of UiO-66-NH_2_ result in an absorption peak redshift to 1300 nm (Fig. 1d, red line). However, at a higher reaction temperature of 75 °C, the formation of island-on-shell nanostructures resulted in an absorption peak blueshift to 950 nm (Fig. 1d, blue line). These results imply that the maximum absorption of UGs is significantly red-shifted as compared to the island-on-core, island-on-shell nanostructures and other reported gold shells, such as silica@gold nanoshells (800 nm) [40], PGNSs (600–800 nm) [41], PUA (800–810 nm) [15], Pt-MOF@GNSs (800 nm) [16], gold nanoshells (832 nm) [42] and SPIO-HGNS (945 nm) [43] and so on [44–47] (Table S1), rendering UGs a highly desirable photothermal agent imaging and PTT for cancers.

By optimizing formic acid, reaction temperature, and other reaction conditions, including the appropriate mass ratio between UiO-66-NH_2_ and HAuCl_4_, the amount of K_2_CO_3_ and PVP (Figures S2-S5), the generation rate of gold nanoparticles could be controlled to result in *V*_dep_ < *V*_diff_, and we obtained a newfangled surface diffusion growth pattern [48, 49] of gold nanoshells (points → facets → octahedron), which was monitored at different stages by scanning electron microscopy (SEM) and TEM. As shown in Fig. 1e, in the initial stage of the reaction, a few gold seeds were formed on the surface of the octahedral UiO-66-NH_2_ nanoparticle to form “gold points”, and then these gold points continued to accumulate into “gold facets” (ranging from the first face to the seventh) by diffusion along the MOF face, and ultimately formed “gold octahedra”. The solution color, the UV-Vis absorption, and the SEM were used to monitor the growth patterns of gold nanoshells at different reaction times. The growth process of gold nanoshells on the surface of UiO-66-NH_2_ could be observed directly through the color change of the solution, which gradually changed from colorless to oxford-blue in 30 min (Figure S6a). As shown in Figure S6b, a few gold nanoparticles were dotted on each surface of UiO-66-NH_2_ within 5 min. This is followed by the rapid propagation of these “gold points” within the same surface to gradually form the “gold facets” within 10 min. At 15 min, some UiO-66-NH_2_ cores were fully covered with thick gold nanoshells and formed “gold octahedrons”. When the growth time was prolonged to 20 min, a dominant amount of UiO-66-NH_2_ cores were covered with gold nanoshells. In 30 min, nearly all the surfaces of UiO-66-NH_2_ were covered with gold nanoshells. The UV-Vis spectra also displayed a gradual increase of absorption peak at 1300 nm in 30 min (Figure S6c), corroborating the observations made from SEM.

We next calculated the electric field enhancement (|E|/|E0|) of the island-on-core, core-shell, and island-on-shell nanostructures by using finite difference time domain (FDTD) for simulating the distribution of near-field electromagnetic fields, respectively. From the simulation results (Fig. 1f and Figure S7), the island-on-core nanostructure shows a weak electrostatic field distribution under 1064 nm laser excitation, which is due to the interaction between the thermal effect of the metallic Au itself and the excitation light. It is worth noting that the formation of Au core-shell nanostructure makes the 1064 nm light absorption cross-section increase significantly and exhibits a prominent electric field enhancement relative to the gold sphere, while the localized surface plasmon resonance (LSPR) peak in the absorption spectrum is substantially red-shifted from 600 nm to above 1300 nm. The photoelectric effect of metal Au nanoshells is obvious under electromagnetic wave irradiation in the *z*-axis, and the resistive heat loss is efficiently converted to thermal energy. The simulated results further indicate that the electric field intensity of gold island-on-shell nanostructures slightly decreases and the LSPR peak blue shifts to about 1000 nm, which is due to some of the gold particles growing into the gold island, and the thickness of the overall shell structure decreases, resulting in absorption cross section decreases, which is consistent with the expected results.

**Fig. 2:**
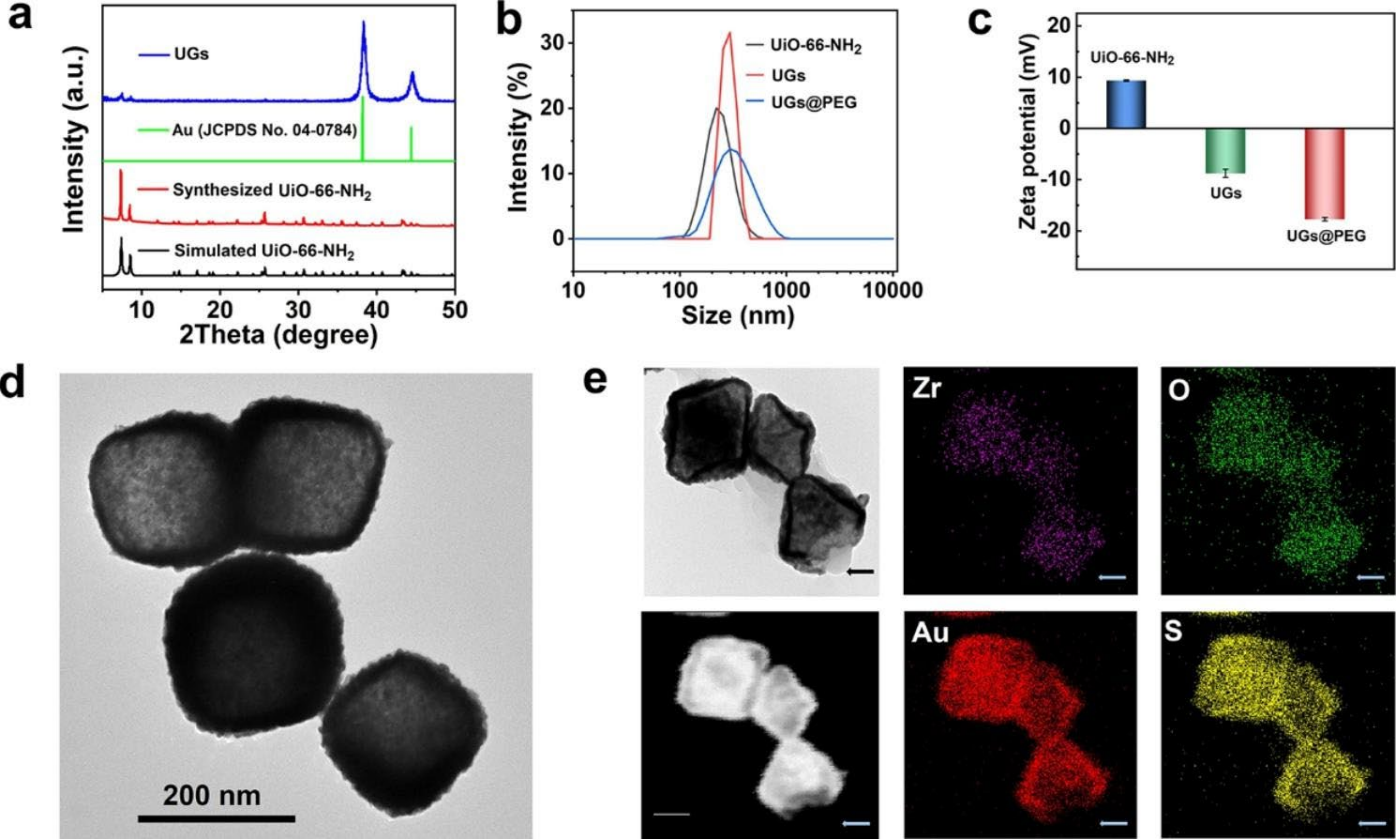
(a) The PXRD patterns of simulated UiO-66-NH_2_, the synthesized UiO-66-NH_2_, Au (JCPDS No. 04-0784), and UGs. (b) The hydrodynamic size distributions and (c) the zeta potentials of UiO-66-NH_2_, UGs, and UGs@PEG in deionized water. (d) The TEM and (e) elemental mapping images of UGs@PEG, scale bar: 100 nm

The powder X-ray diffraction (PXRD) patterns verify that the prepared UiO-66-NH_2_ is isostructural to that reported and the remarkably increased intensity of Au diffraction peak (ca. 2θ = 38°) of the formed UGs verified the formation of gold nanoshells. The similar PXRD profiles before and after gold nanoshell wrapping indicate the maintenance of the crystallinity and structural integrity of UiO-66-NH_2_ throughout the coating process (Fig. 2a). To improve the biocompatibility and physiological stability of UGs, we further modified UGs with polyethylene glycol thiol (PEG-SH) to give UGs@PEG [15].

The average hydrodynamic sizes of UiO-66-NH_2_, UGs, and UGs@PEG were 216 nm, 271 nm, and 278 nm, respectively (Fig. 2b). As depicted in Fig. 2c, the zeta potentials of UiO-66-NH_2_ (9.35 mV), UGs (− 8.78 mV), and UGs@PEG (− 17.73 mV) further demonstrated that the gold nanoshells and PEG-SH were deposited on UiO-66-NH_2_. The broad vision TEM images of UGs@PEG distinctly revealed that the gold nanoshells were coated on the surface of UiO-66-NH_2_ with high efficiency and yield with the retention of morphological shapes of UiO-66-NH_2_ (Fig. 2d). The elemental mapping of UGs@PEG showed that its main elemental compositions were Au, S, Zr, O, and N (Fig. 2e and Figure S8). Notably, for the uncovered part of UiO-66-NH_2_ (indicated by the arrows), Au and S elements were simultaneously absent. These observations not only reflected the growth pattern (points → facets → octahedron) of gold nanoshells but also indicated that SH functionality in PEG-SH induces the formation of Au-S bond [50, 51].

**Fig. 3:**
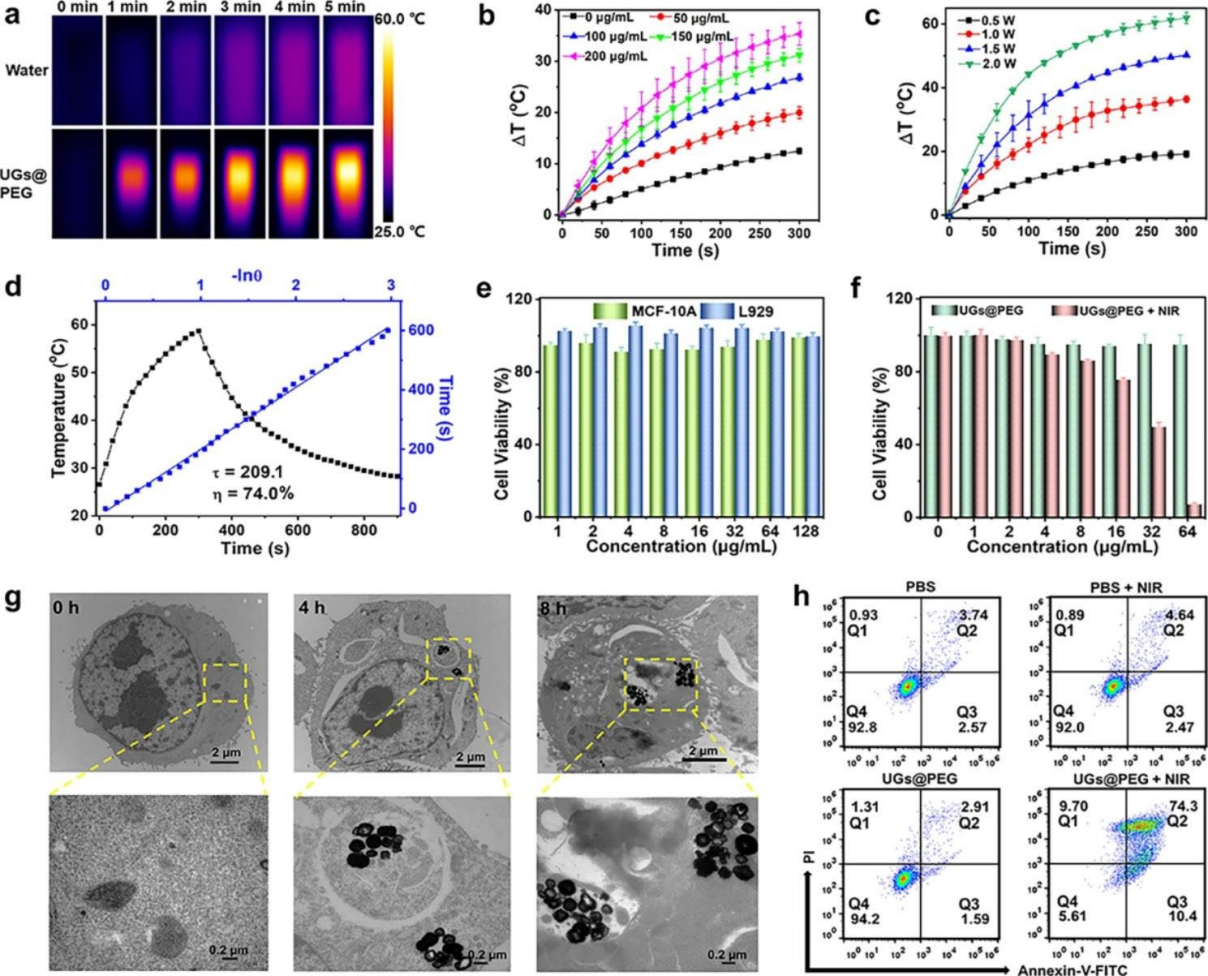
(a) The thermographic images of water and UGs@PEG (200 *µ*g mL^− 1^) with laser irradiation for 5 min (1064 nm, 1.0 W cm^− 2^). (b) In vitro temperature rising curves of UGs@PEG at different concentrations (0, 50, 100, 150 and 200 *µ*g mL^− 1^) and (c) at different irradiation power (0.5, 1.0, 1.5 and 2.0 W cm^− 2^) (n = 3). (d) Linear time vs.–ln(θ) was obtained from the first cooling period. (e) The cell viability of MCF-10 A and L929 cells treated with UGs@PEG at different concentrations of (1, 2, 4, 8, 16, 32, 64, and 128 *µ*g mL^− 1^) for 24 h. (f) The 4T1 cell viability after being treated with UGs@PEG with or without NIR light at different concentrations (0, 1, 2, 4, 8, 16, 32, and 64 *µ*g mL^− 1^). (g) The bio-TEM images for cell uptake of UGs@PEG (50 *µ*g mL^− 1^) in 4T1 cells at 0, 4, and 8 h, scale bar: 2 *µ*m (top) and 0.2 *µ*m (bottom). (h) The cell apoptosis analysis results of 4T1 cells after different treatments obtained by flow cytometry (Q1: necrotic cells; Q2: secondary necrotic/late apoptotic cells; Q3: early apoptotic; Q4: vital cells)

Encouraged by the preeminent NIR-II absorption ability of UGs@PEG, its photothermal conversion performance in aqueous solution upon a 1064 nm laser irradiation was assessed by a digital NIR photothermal imaging system. The results indicated that UGs@PEG exhibited an irradiation time-, concentration-, and power-dependent temperature increase. As shown in Fig. 3a, the NIR-II thermal imaging indicated that the aqueous solution with UGs@PEG showed an intense temperature increase from 25.0 to 59.3 °C after 5 min, while the deionized water barely showed any response to the laser. The UGs@PEG aqueous solution also showed a concentration-dependent temperature change upon laser exposure (1.0 W cm^− 2^, 5 min) and the temperature gradually increases with the increase of concentrations under given irradiation time and power (Fig. 3b). The temperature also increases with the augment of irradiation power (Fig. 3c).

To evaluate the photothermal stability and reliability, we also conducted four consecutive cycles of UGs@PEG solutions (200 *µ*g mL^− 1^) from heating to cooling by 1064 nm laser irradiation (1.0 W cm^− 2^) and recorded the temperature every 20 s. As shown in Figure S9, there is no significant attenuation observed for each cycle, suggesting that UGs@PEG can act as highly stable photothermal agents for cancer therapy. The photothermal conversion efficiency (*η*) of the UGs@PEG is as high as 74.0% (Fig. 3d), which is marginally higher than the reported gold nanoshells, such as silica@gold nanoshells (8.3%), [40] PGNSs (22.4%), [45] Pt-MOF@GNSs (38.6%), [16] gold nanoshells (45.0%), [42] PUA (58.7%), [15] and SPIO-HGNS (60.0%) [43] (Table S1).

Encouraged by the promising photothermal properties of the UGs@PEG, the therapeutic effect was evaluated at the cellular level. Initially, the required cells were cultured to keep the normal growth state. The cytotoxic effect of the samples was analyzed by assessing the viability of MCF-10 A and L929 cells treated with UGs@PEG at different concentrations (1, 2, 4, 8, 16, 32, 64, and 128 *µ*g mL^− 1^) for 24 h using the standard MTT assay. As depicted in Fig. 3e, almost all MCF-10 A and L929 cells survived after incubation with UGs@PEG with a concentration below 128 *µ*g mL^− 1^, indicating its good biocompatibility. The cell growth inhibitory effect of UGs@PEG on 4T1 was subsequently evaluated. As shown in Fig. 3f, the toxicity of UGs@PEG + NIR is dose-dependent. Compared to the UGs@PEG group, the UGs@PEG + NIR group showed an exceptional killing efficiency of tumor cells with more than 95% cell death due to the UGs-based PTT effect.

The bio-TEM imaging for cellular uptake of UGs@PEG (50 *µ*g mL^− 1^) within 4T1 cells was utilized to confirm the cell internalization ability of UGs@PEG. As shown in Fig. 3g, it is clear that a portion of UGs@PEG was accumulated in the endosomes after 4 h incubation, and increased slightly after 8 h. These results revealed that UGs@PEG could be effectively uptaken by 4T1 cells, indicating that UGs@PEG could enter the tumor cells smoothly to elicit a therapeutic role.

To better measure the therapeutic effect of NIR laser-mediated PTT, after incubation with PBS or UGs@PEG for 24 h, 4T1 cells were divided into four groups: PBS (i), PBS + NIR (ii), UGs@PEG (iii), and UGs@PEG + NIR (iv). Then groups ii and iv were further treated with NIR light for 5 min followed by double-staining with Annexin-V/PI to verify the type of cell death by flow cytometry. Early apoptotic cells are those positive for Annexin V alone, necrotic cells are those positive for PI alone, and secondary necrotic and/or late apoptotic cells are both positive for Annexin V plus PI. As shown in Fig. 3h, quantitative flow cytometric results showed that the PBS, PBS + NIR, and UGs@PEG groups had favorable biocompatibility, while UGs@PEG + NIR dramatically decreased the cell viability to 5.61%. 4T1 cells mainly underwent secondary necrosis and/or late apoptosis accompanied by slight early apoptosis and necrosis.

To visually observe the antitumor activity of different groups in vitro, the 4T1 cells were stained with calcein-AM for living cells as green and PI for dead cells as red. As shown in Figure S10, the PBS, PBS + NIR and UGs@PEG groups showed no obvious damage to the cells, while almost all the cells died when treated with UGs@PEG + NIR. These results mirror those of the MTT experiments, which further demonstrated that the UGs@PEG in combination with NIR laser irradiation showed a preeminent anti-cancer efficacy.

Biocompatibility is considered the prerequisite for the application of nanoparticles in vivo. As shown in Figure S11, with the increase of UGs@PEG concentration in 0–300 *µ*g mL^− 1^, no visual red color was observed in the hemocompatibility test, indicating that UGs@PEG did not cause apparent hemolysis under 300 *µ*g mL^− 1^.

PAI is an emerging modality that generates high-definition volumetric images of tissue by measuring light-induced sound waves from its optically absorbing structures [52–54]. Due to the exceptional absorbance of UGs@PEG in the NIR-II window with the merits of deeper tissue penetration, their photoacoustic performance in vitro was explored. Photoacoustic images of aqueous UGs@PEG suspensions in deionized water at various concentrations (0, 32, 63, 125, 250, and 500 *µ*g mL^− 1^) in the front and cross-section of photoacoustic tubes under 1064 nm laser can be observed (Figure S12a), indicating a promising PAI capability of UGs@PEG in NIR-II windows. As shown in Figure S12b, there is a good linear relationship between PAI value and UGs@PEG concentration in the range of 0–500 *µ*g mL^− 1^. The photoacoustic intensity of UGs@PEG aqueous suspensions is significantly higher than that of deionized water and increases notably with their concentrations under 1064 nm lasers, which is consistent with its absorption spectrum.

**Fig. 4:**
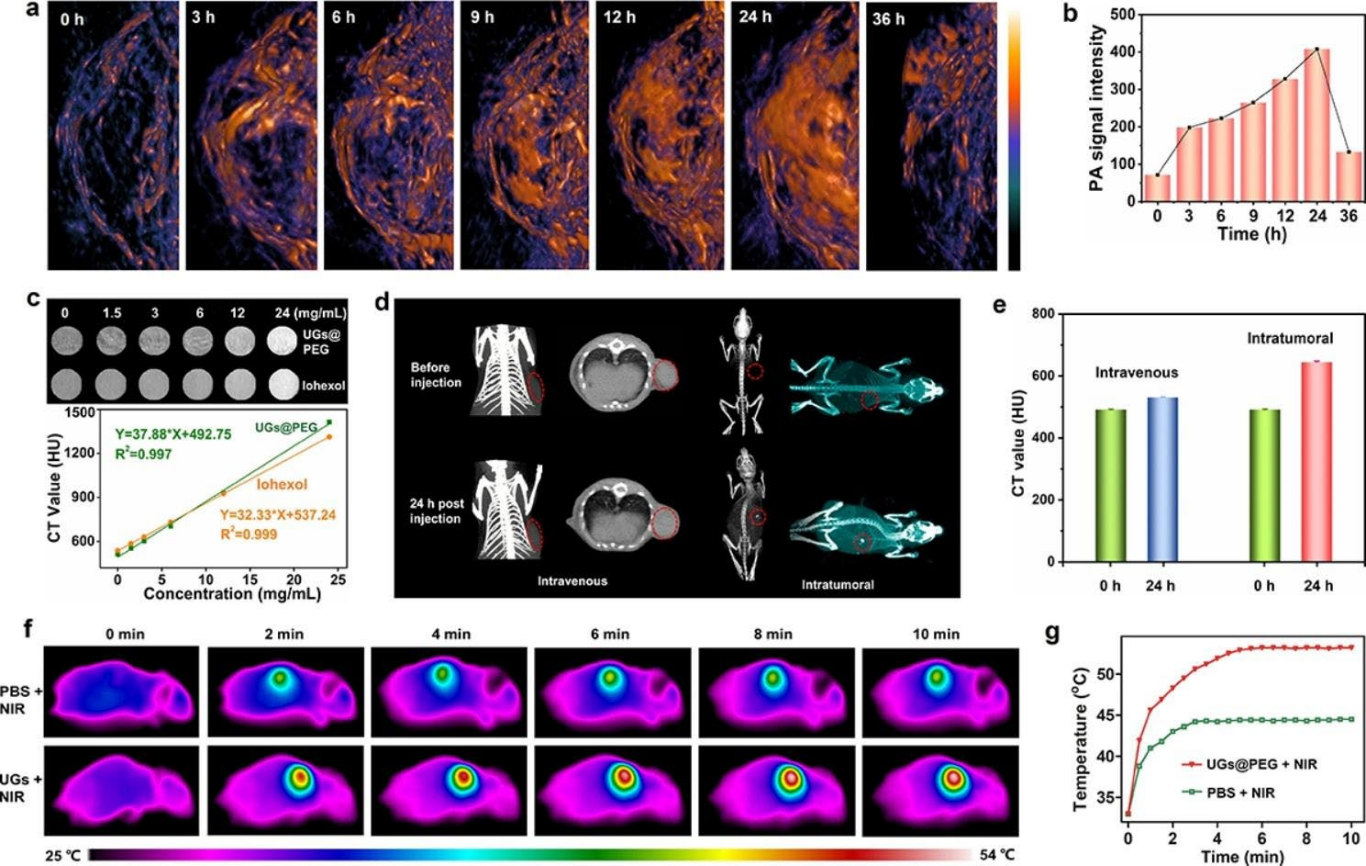
(a) In vivo PAI of the visceral mice and tumor sites under 1064 nm lasers after intravenous injection at different time intervals (0, 3, 6, 9, 12, 24, and 36 h). (b) The PAI imaging intensity at different time intervals (0, 3, 6, 9, 12, 24, and 36 h). (c) In vitro CT images of UGs@PEG with different concentrations (0, 1.5, 3, 6, 12, and 24 mg mL^− 1^) and CT Hounsfield unit (HU) plot. (d) In vivo CT images for 24 h. (e) The CT value of tumor-bearing mice before and after intravenous (i.v.) and intratumoral (i.t.) injections of UGs@PEG. (f) In vivo representative photothermal images of tumor-bearing mice under 1064 nm laser irradiation for 10 min after injection with PBS and UGs@PEG. (g) The corresponding heating curves in the tumor regions of mice treated with PBS and UGs@PEG

To maximize the in vivo therapeutic effect, the tumor peak accumulation time of UGs@PEG was also studied. 4T1 tumor-bearing mice were intravenously (i.v.) administrated UGs@PEG. The results showed that PAI signals in tumor tissue enhanced gradually with the increasing injection time (0, 3, 6, 9, 12, 24, and 36 h), attributed to the enhanced permeability and retention (EPR) effect. The PA signal reached a maximum of around 24 h post-injection and almost disappeared after 36 h (Fig. 4a-b), indicating that nanomaterials could be metabolized gradually in vivo and do not accumulate in blood vessels or other sites after the treatment. All these above results showed that UGs@PEG have good potential for NIR-II PA imaging to guide tumor therapy.

CT has been widely used as a powerful diagnostic tool in clinics because it can provide high-resolution 3D tomography of the anatomic structure based on the distinctive X-ray absorptions between different tissues. Au nanoparticles have been used as contrast agents for CT imaging due to their better X-ray attenuation than normal contrast agents [55, 56]. To evaluate the potential of UGs@PEG as an effective CT contrast agent, CT imaging was assessed using the same concentrations gradient of UGs@PEG and the clinically available iohexol, respectively. As shown in Fig. 4c, the CT signals brightened gradually as the concentration of UGs@PEG increased (0, 1.5, 3, 6, 12, and 24 mg mL^− 1^). The Hounsfield unit (HU) value displayed a well-correlated linear relationship with the UGs@PEG concentration. Noticeably, UGs@PEG exhibits a higher HU value than iohexol, indicating that UGs@PEG holds enormous potential for CT imaging.

Owing to the good CT performance in vitro, we further validated the suitability of UGs@PEG for in vivo CT imaging. As such, an investigation was conducted on the breast cancer tumor-bearing mice after i.v. and intratumoral (i.t.) injections of UGs@PEG. As shown in Fig. 4d-e, the rise of CT signals was observed after i.v. injection and significantly higher CT signals were observed after i.t. injection. Compared with pre-injection, signals after 24 h post-injection was again evidence for the high accumulation of UGs@PEG at the tumor site, which will overcome the drawbacks of clinically used small iodinated molecules with short blood-retention time.

In vivo temperature rising and photothermal imaging (PTI) of UGs@PEG were studied on 4T1 tumor-bearing mice. Twenty mice are randomly divided into four groups: PBS (i), PBS + NIR (ii), UGs@PEG (iii), and UGs@PEG + NIR (iv). A continuous-wave NIR-II window laser was used to irradiate the solid tumors for 10 min (1064 nm, 1.0 W cm^− 2^), and the temperature of the tumors was monitored by a thermal camera. As expected in Fig. 4f-g, the final temperature of the UGs@PEG + NIR group is close to 53.1 °C in 5 min with an obvious PTI property. In contrast, the mice injected with PBS represented only an increase to 44.3 °C in the tumor area during the entire irradiation process, suggesting that the photothermal response of nanomaterials is mainly generated by the gold nanoshells.

Based on the previous imaging in vivo results, at 24 h after the intravenous injection, 4 mice were sacrificed, and the main organs and tumor were excised and weighed, the contents of Au element in the main organs and tumor were examined by ICP-MS. The results revealed that the accumulation of UGs@PEG in the tumor was comparable to the spleen, higher than most major organs except the liver (Figure S13).

**Fig. 5:**
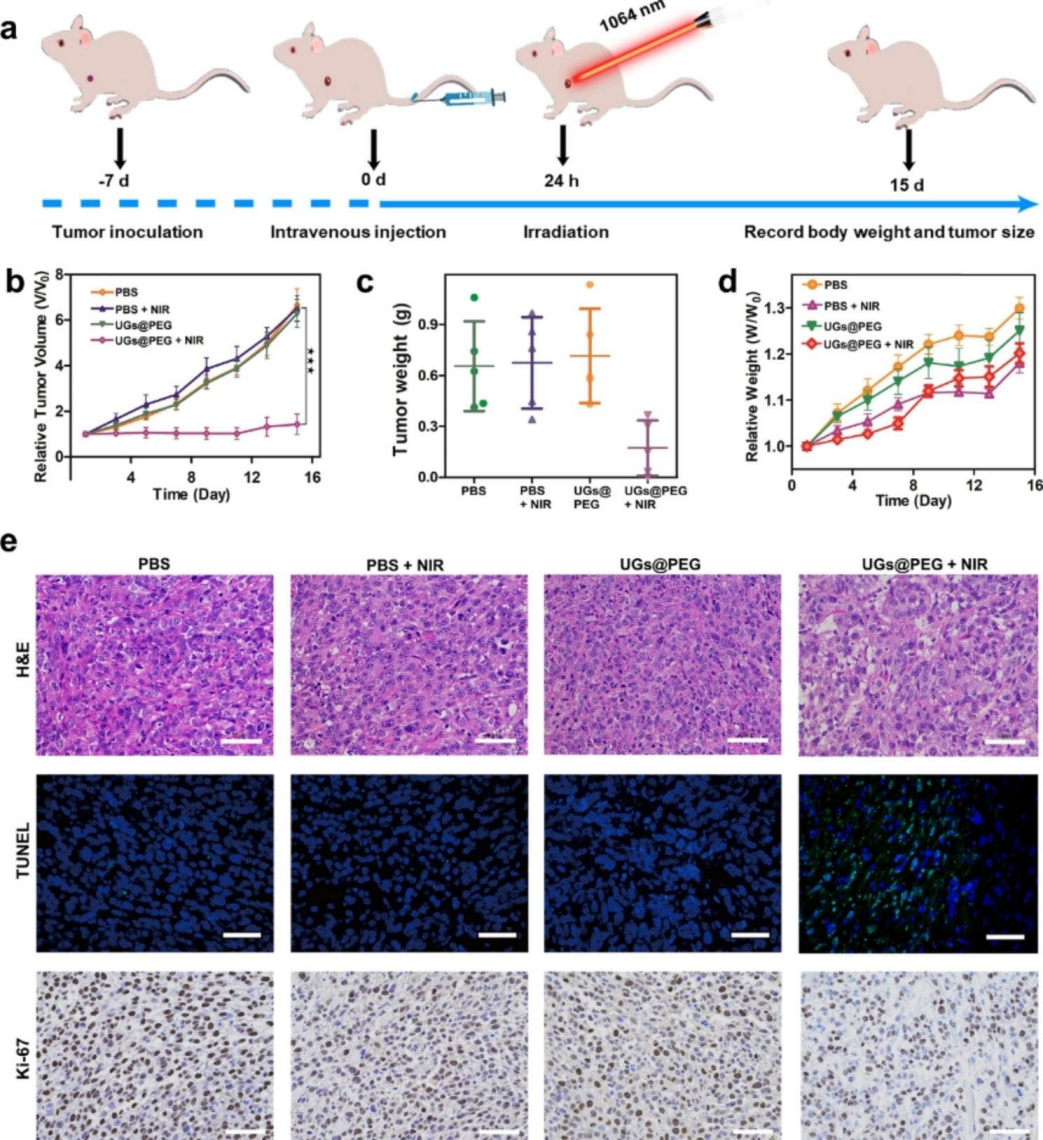
(a) Schematic illustration of therapeutic effect studies in vivo. (b) The average change of tumor volume and (c) tumor weight of different treatment groups on the 15th day. Asterisks (*) represent a significant difference compared with the UGs@PEG + NIR group. *p < 0.05, **p < 0.01, and ***p < 0.001. (d) Bodyweight changes in mice from different treatment groups for 15 days. (e) H&E (1st row), TUNEL (2nd row ), and Ki-67 (3rd row) staining of the dissected tumor tissues after different treatments. Scale bar: 50 *µ*m

Benefiting from the results of multimodal imaging and ICP-MS analyses, the in vivo antitumor activity of UGs@PEG was further studied using Balc/c mouse models. When the tumor volume reached 100 mm^3^ after inoculation with 4T1 cells for approximately 7 days, 28 mice are randomly divided into four groups: PBS (i), PBS + NIR (ii), UGs@PEG (iii), and UGs@PEG + NIR (iv). At 24 h after the intravenous injection, a continuous-wave 1064 nm laser was used to irradiate groups ii and iv for 10 min (1.0 W cm^− 2^, Fig. 5a), and the tumor volumes of mice were recorded during the therapeutic process (Figure S14). The results indicated that tumors in the PBS, PBS + NIR, and UGs@PEG groups grew rapidly, presumably due to the good biocompatibility and low toxicity of UGs@PEG itself. By comparison, UGs@PEG + NIR could prominently inhibit tumor growth ascribed to its PTT effect. All the tumor-bearing mice in the treatment groups were sacrificed after 15 days of treatment, and the tumors were collected and photographed (Fig. 5b). At the same time, the average change of tumor weight of different treatment groups was calculated (Fig. 5c), which pointed to the exceptional tumor inhibition ability of UGs@PEG. The body weight of mice was recorded during the therapeutic process (Fig. 5d), which showed that UGs@PEG + NIR could inhibit tumor growth and finally achieve the goal of tumor treatment.

The outstanding antitumor effects were also confirmed by the hematoxylin and eosin (H&E) and terminal-deoxynucleotidyl transferase-mediated nick end labeling (TUNEL) staining, which showed the nucleus was significantly reduced (Fig. 5e, 1st row) and there was strong green fluorescence (Fig. 5e, 2nd row), indicating that UGs@PEG can induce tumor apoptosis and achieve the purpose of tumor therapy. In addition, Ki-67 antibody staining was used to measure the proliferation activity in vivo. Compare with the other three groups, UGs@PEG + NIR group had a strong inhibitory effect on cell proliferation (Figs. 5e, 3rd row). Finally, the visceral damage in mice was assessed by the H&E staining, and the results showed that the major organs of tumor-bearing mice in various treatment groups displayed no observable damage or inflammation after the treatment, indicating a high histocompatibility and biosafety of UGs@PEG (Figure S15).

## Conclusion

In this work, we reported a one-pot seed-mediated cascade process to obtain island-on-core, core-shell, and island-on-shell gold nanostructures. The seed-mediated cascade growth process can be modulated by adjusting the proportion of formaldehyde and formic acid, as well as the reaction temperature, which allows gold nanoshells to form through a well-oriented and controllable diffusion growth pattern (points → facets → octahedron). Thanks to the outstanding photothermal conversion efficiency (74.0%) in NIR-II, the gold nanoshells function as a multi-modal platform by showing PTI-/PAI-/CT- guided NIR-II PTT for the efficient suppression of tumors, holding great promises toward clinical translations. The kinetically and thermodynamically controlled one-pot seed-mediated successive approach reported herein may be extended to the synthesis of other core-shell superstructures, such as silver, palladium, and platinum, on a diverse array of morphologically attractive NMOFs, such as ZIF-8, producing efficient photothermal conversion materials with broad applications.

## Electronic supplementary material

Below is the link to the electronic supplementary material.

Supplementary Material 1: Supporting information: Details of the experimental procedures and supplementary results (PDF). Additional TEM images; UV-Vis and EDS spectra; photothermal stability test; living and dead staining images; hemolysis analysis; the tumor volumes of mice; hematoxylin and eosin (HE) staining images; additional table for comparison of UV-Vis absorption, photothermal conversion efficiencies of reported gold nanoshells in PDF

## Data Availability

All data generated or analyzed during this study are included in this published article and additional information.
