# Supplementary material for: Kinetically and thermodynamically controlled one-pot growth of gold nanoshells with NIR-II absorption for multimodal imaging-guided photothermal therapy
Source: J Nanobiotechnology. 2023 Apr 28;21:138. doi: 10.1186/s12951-023-01907-1 (PMC10141956; doi:10.1186/s12951-023-01907-1)
Supplement: Supplementary file 1 — Supplementary Material 1: Supporting information: Details of the experimental procedures and supplementary results (PDF). Additional TEM images; UV-Vis and EDS spectra; photothermal stability test; living and dead staining images; hemolysis analysis; the tumor volumes of mice; hematoxylin and eosin (HE) staining images; additional table for comparison of UV-Vis absorption, photothermal conversion efficiencies of reported gold nanoshells in PDF [file 12951_2023_1907_MOESM1_ESM.docx]

**Supporting Information**

**Kinetically and Thermodynamically Controlled One-pot Growth of Gold Nanoshells with NIR-II Absorption for Multimodal Imaging-Guided Photothermal Therapy**

Ming Chen^1^*, Xiao-Tong Chen^2^, Lian-Ying Zhang^2^, Wei Meng^2^, Yong-Jian Chen^2^, Ying-Shan Zhang^2^, Zhi-Cong Chen^2^, Hui-Min Wang^2^, Chun-Mei Luo^1^, Xiu-Dong Shi^2^, Wen-Hua Zhang^3^, Mao-Sheng Wang^1^ and Jin-Xiang Chen ^2^*

**
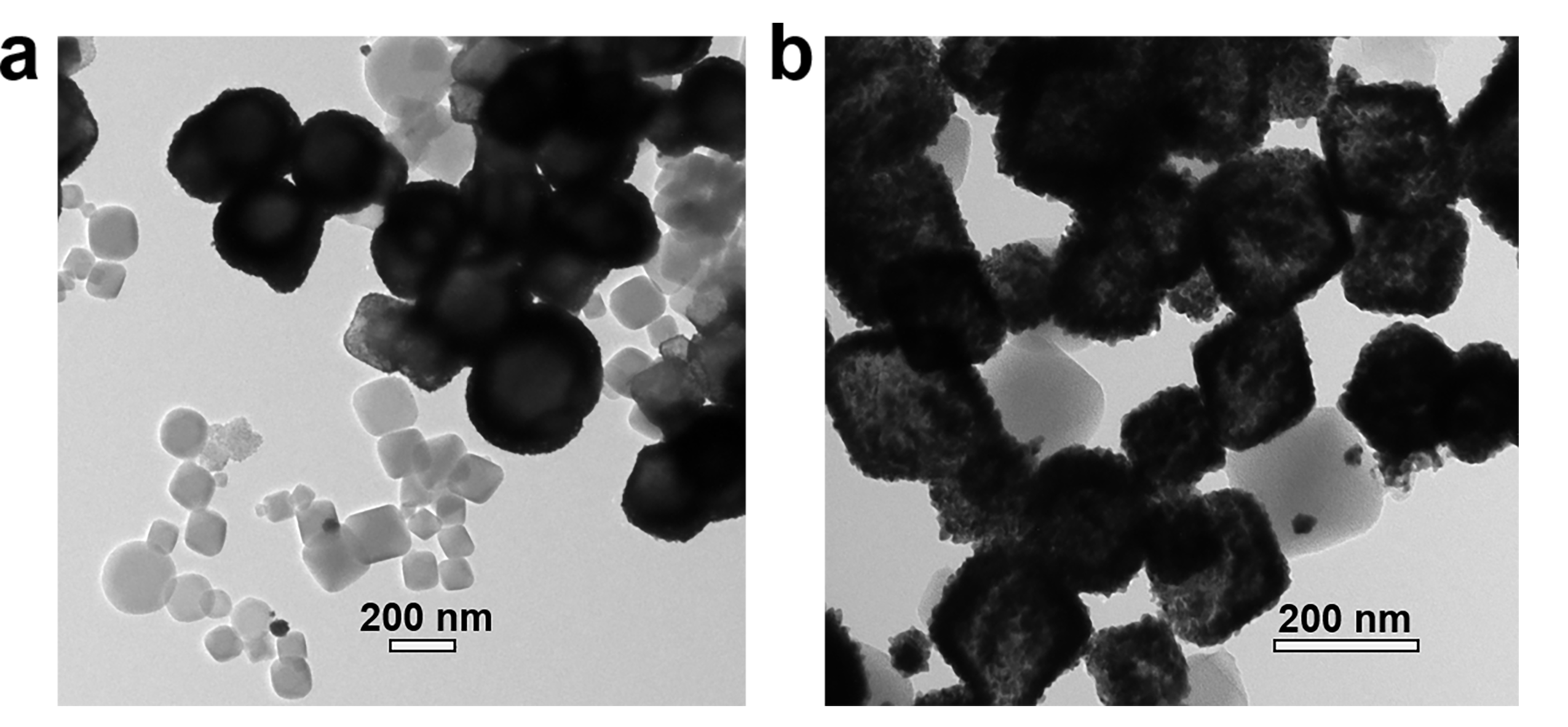
**

**Figure S1** The TEM images of UGs with the ratio of formic acid to formaldehyde being 0.06% at 0 °C (a) and 50 °C (b), show that UGs form more quickly at a high temperature. nanoparticles with the ratio of formic acid to formaldehyde being 0.06% at 0 °C (a) and 50 °C (b), respectively.

**
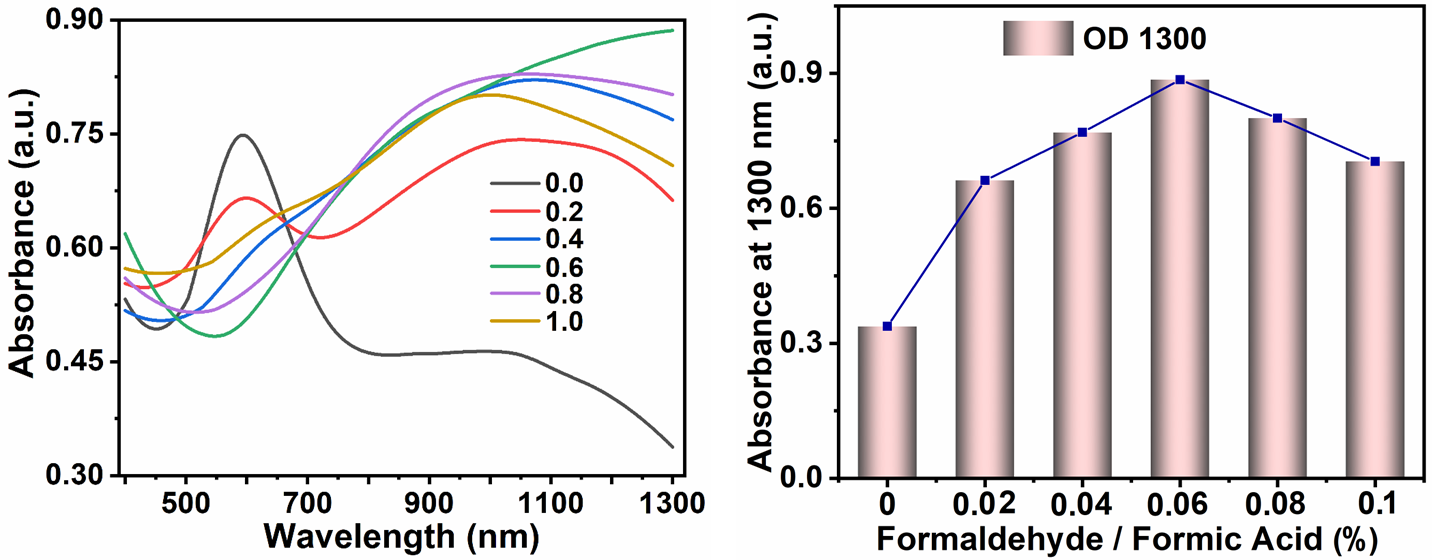
**

**Figure S2** The corresponding UV-Vis absorbance spectra of UGs aqueous solution in 200 *μ*L reductant (formic acid/formaldehyde (*v*/*v*)% = 0%, 0.02%, 0.04%, 0.06%, 0.08%, 0.1%).

**
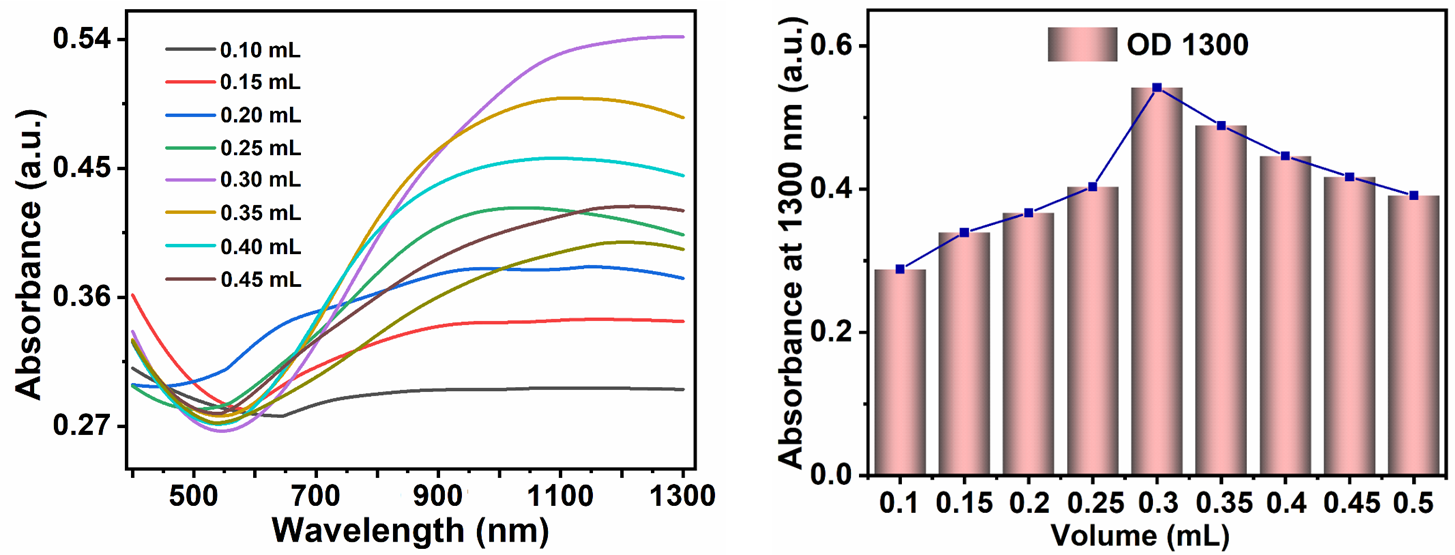
**

**Figure S3** The corresponding UV-Vis absorbance spectra of UGs aqueous solution with different volumes (0.10, 0.15, 0.20 0.25, 0.30, 0.35, 0.40, 0.45, and 0.50 mL) of HAuCl_4_ (10 mg mL^−1^).


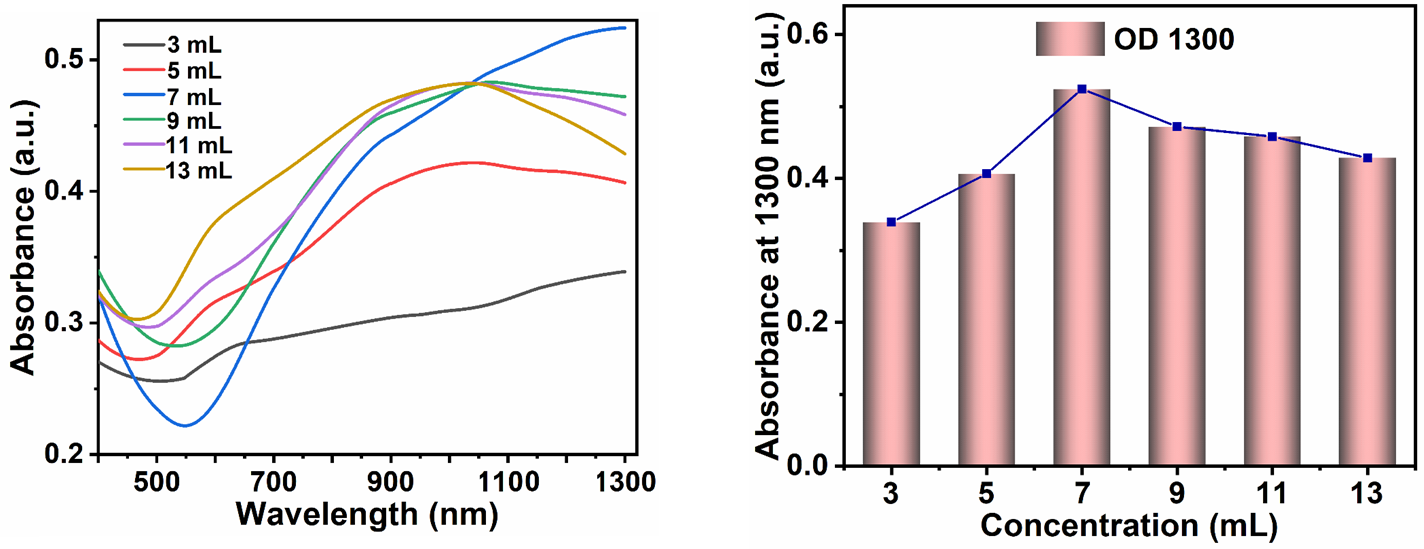


**Figure S4** The corresponding UV-Vis absorbance spectra of UGs aqueous solution at the different volumes (3, 5, 7, 9, 11, and 13 mL) of 3 mg mL^−1^ K_2_CO_3_.


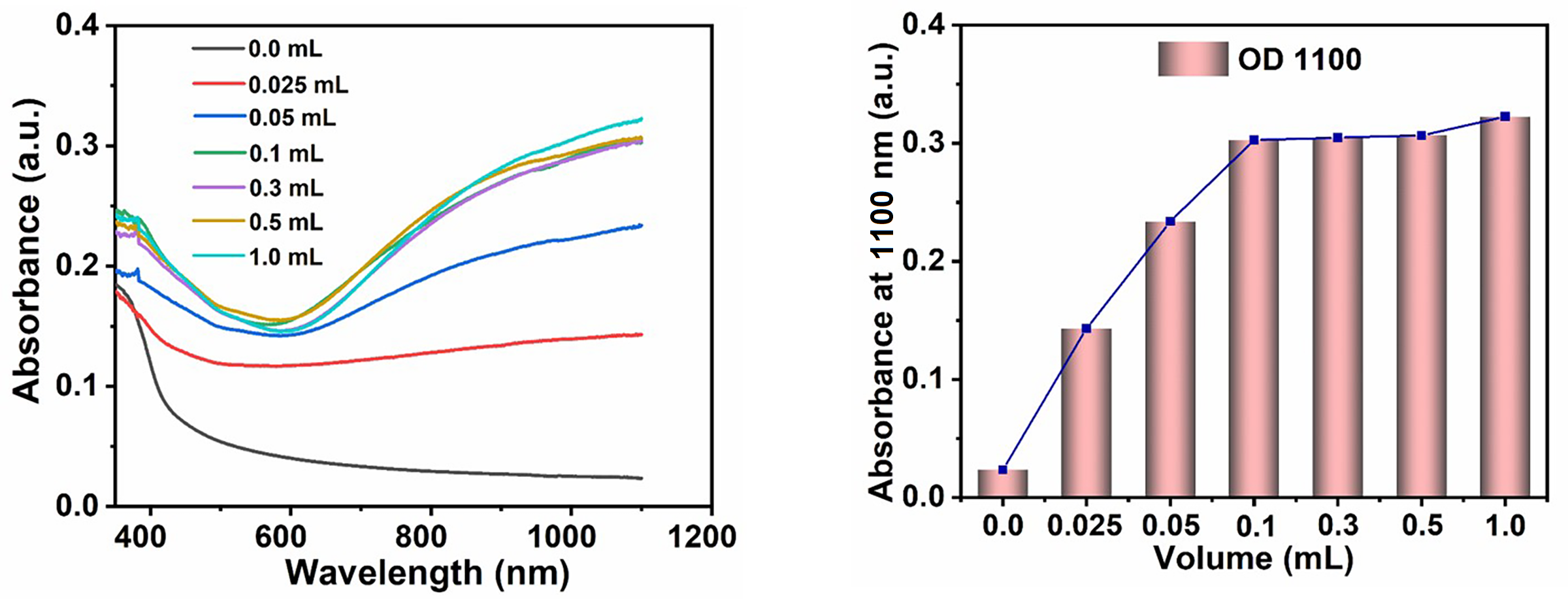


**Figure S5** The corresponding UV-Vis absorbance spectra of UGs aqueous solution at different volume (0, 0.025, 0.05, 0.1, 0.3, 0.5, and 1.0 mL) of 1.0 mg mL^−1^ PVP.


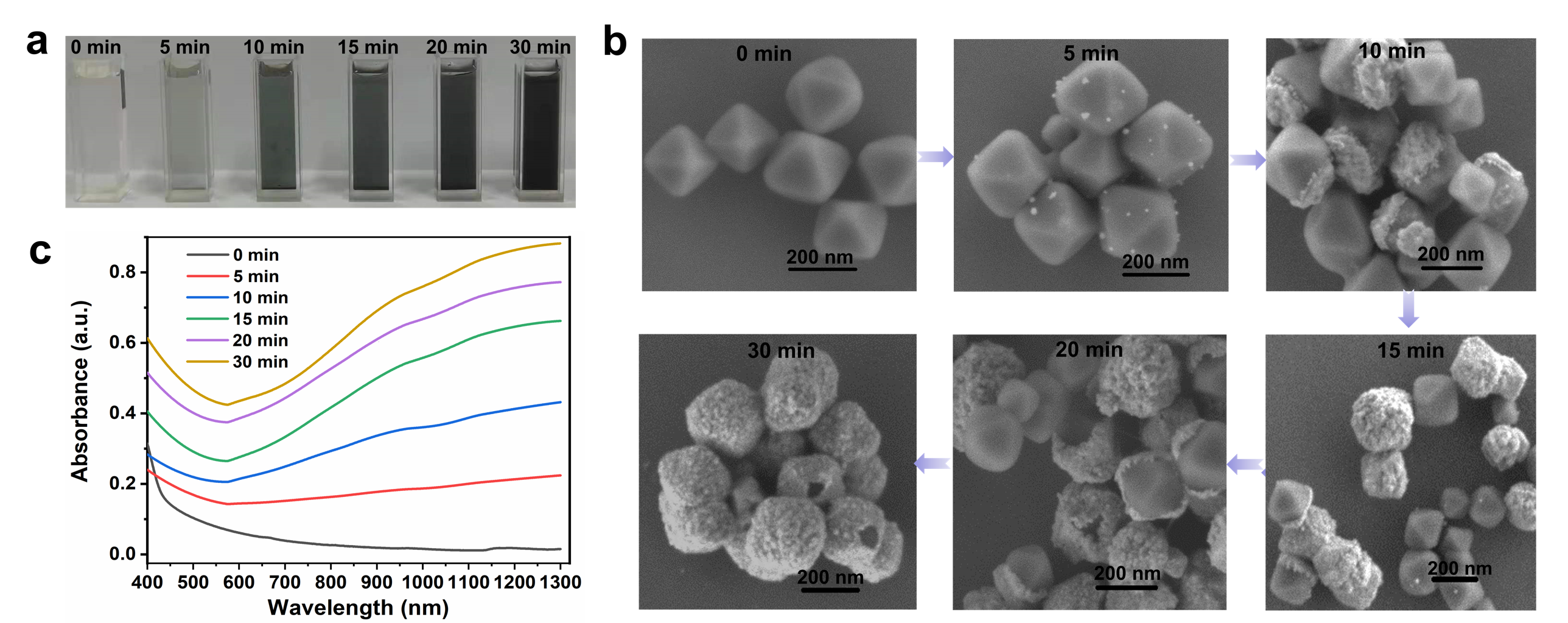


**Figure S6** (a) The aqueous solution color change during the UGs formation process. (b) The SEM images, and (c) the UV-Vis for the formation process of UGs at different reaction times (0, 5, 10, 15, 20, 30 min).


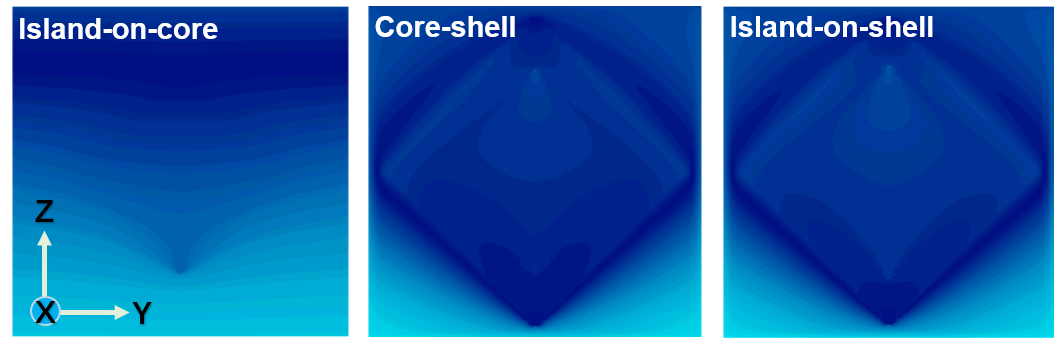


**Figure S7** Electric field distributions of the island-on-core, core-shell, and island-on-shell nanostructures with an incidence of 1064 nm laser at the x-direction.


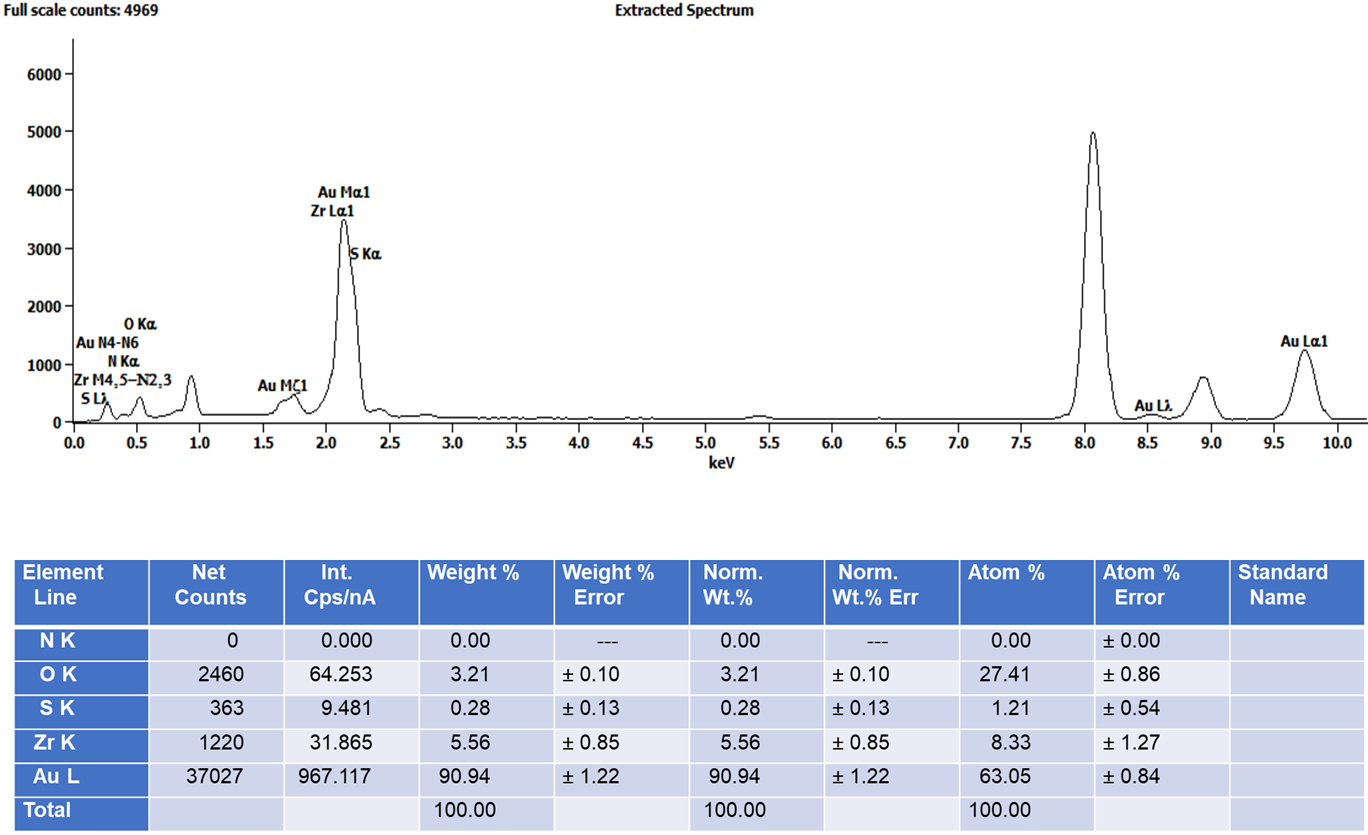
**Figure S8** The EDS spectrum of UGs and analysis results for UGs.


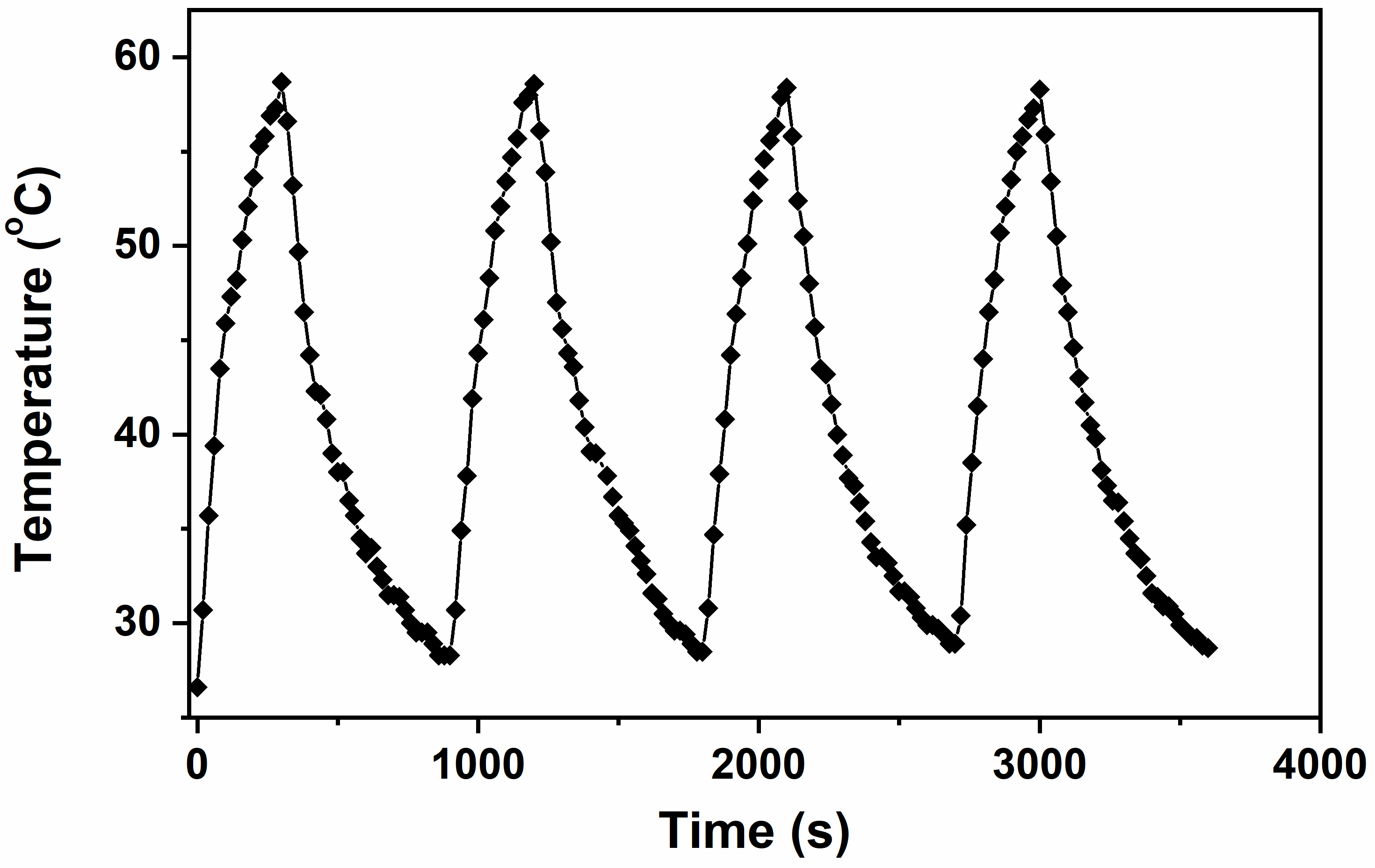


**Figure S9** The photothermal stability test of UGs with four heating/cooling cycles under 1064 nm laser irradiation (200 *μ*g mL^−1^, 1.0 W cm^−2^).


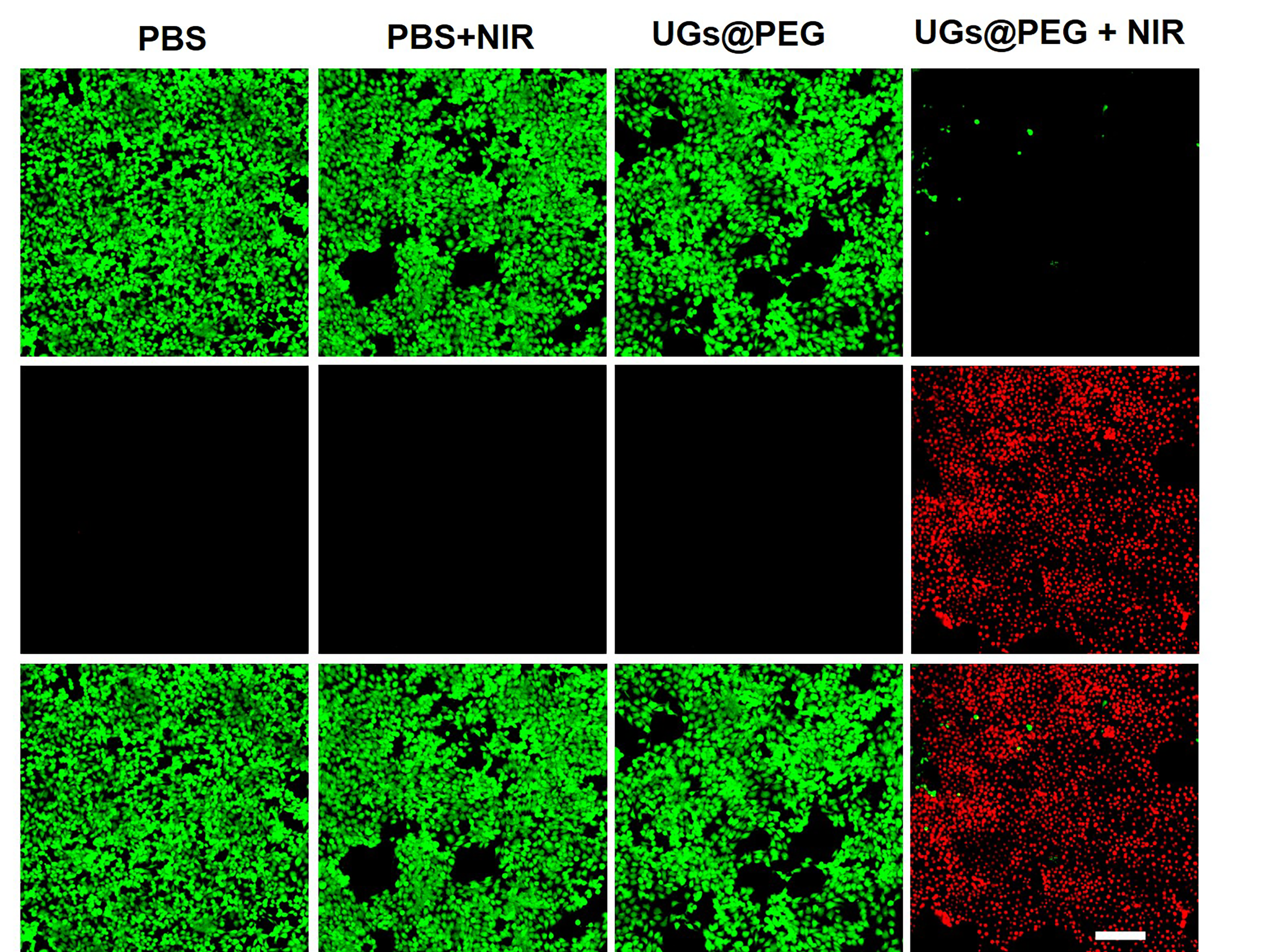


**Figure S****10** The fluorescence images of 4T1 cells upon living (green) and dead (red) staining after various treatments. A laser irradiation (1064 nm) power at 1.5 W cm^−2^ for 3 min was employed. Scale bar: 100 *μ*m.


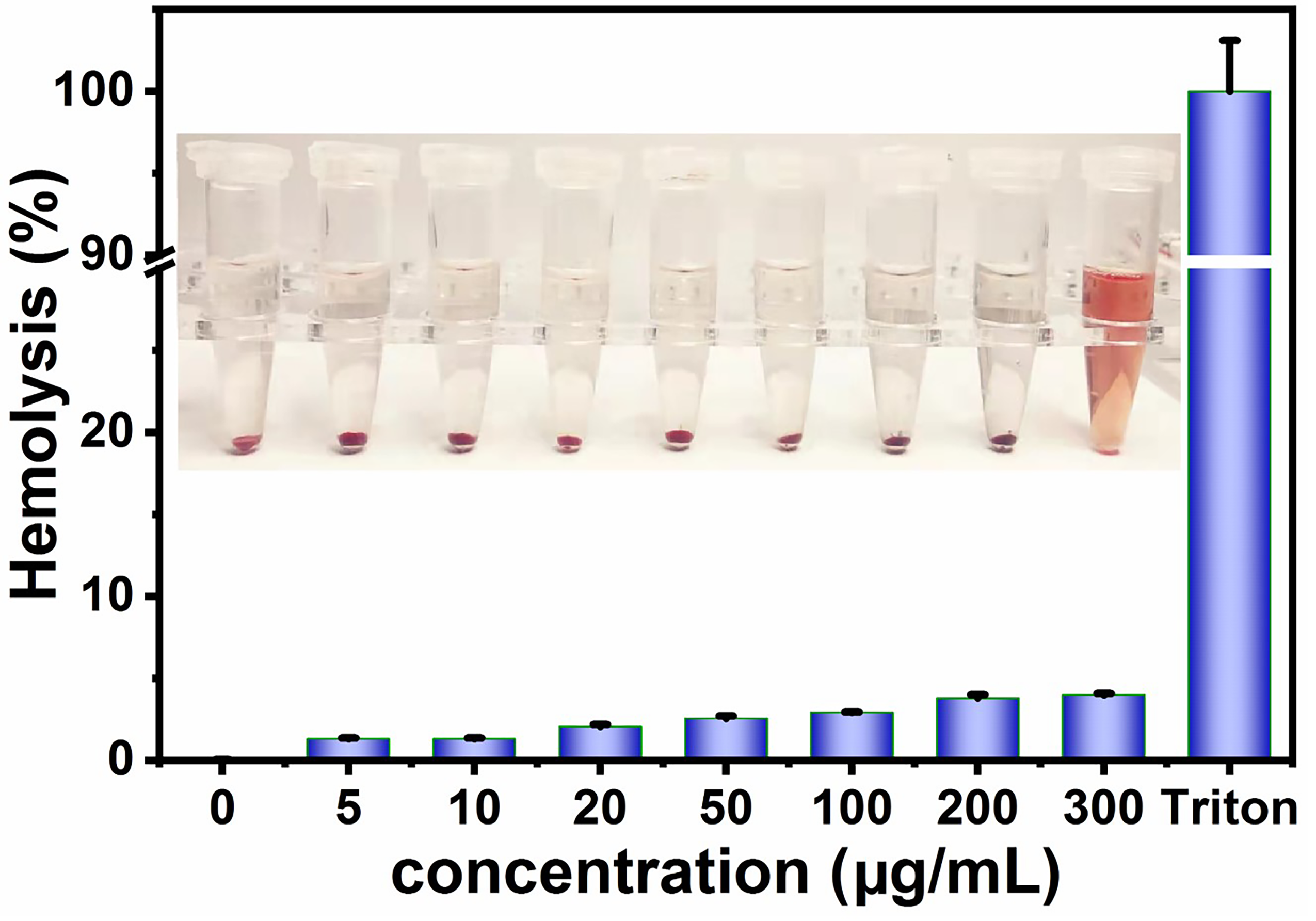


**Figure S11** The hemolysis analysis of UGs@PEG.


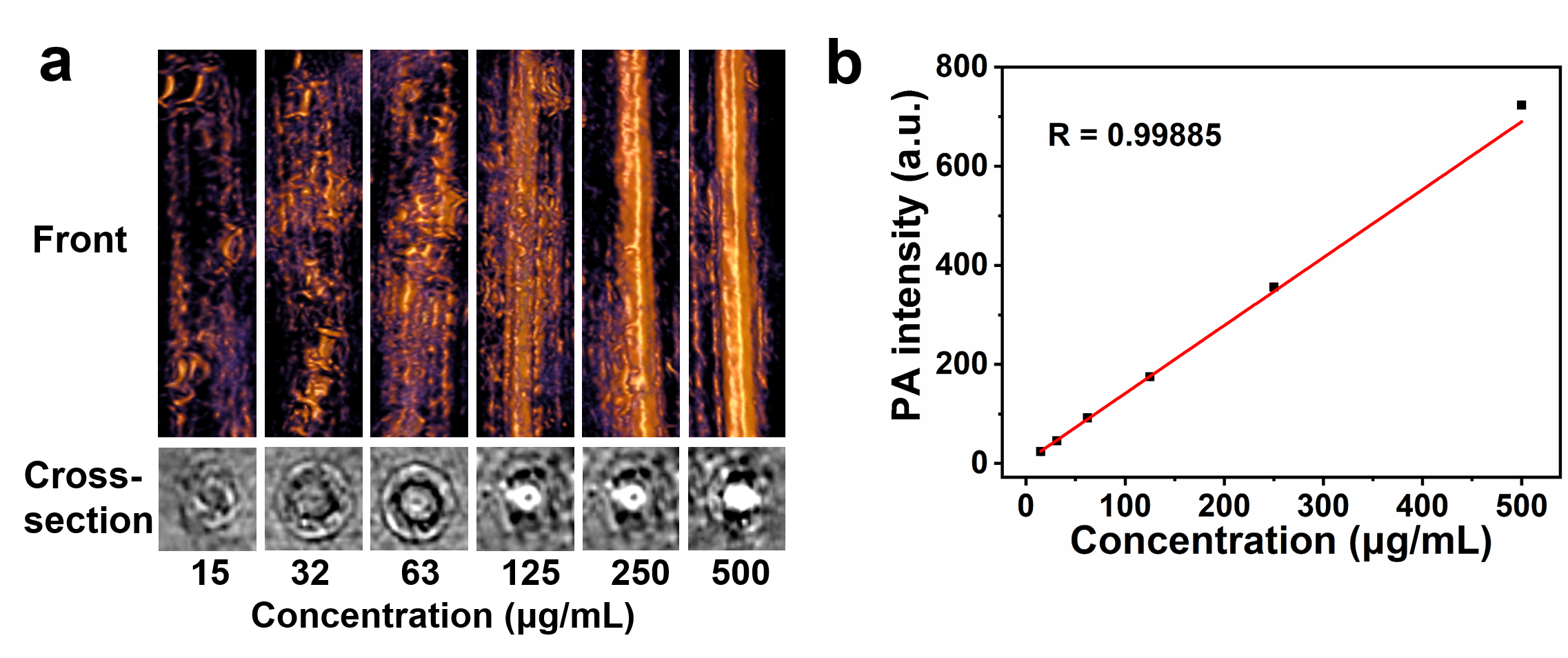


**Figure S12** (a) The photoacoustic imaging (PAI) of deionized water and UGs@PEG aqueous suspensions with different concentrations (15, 32, 63, 125, 250, and 500 μg mL^−1^) in the front and cross-section of photoacoustic tubes under 1064 nm lasers. (b) The photoacoustic signals of deionized water and UGs@PEG aqueous suspensions (15, 32, 63, 125, 250, and 500 μg mL^−1^) under 1064 nm lasers.

**
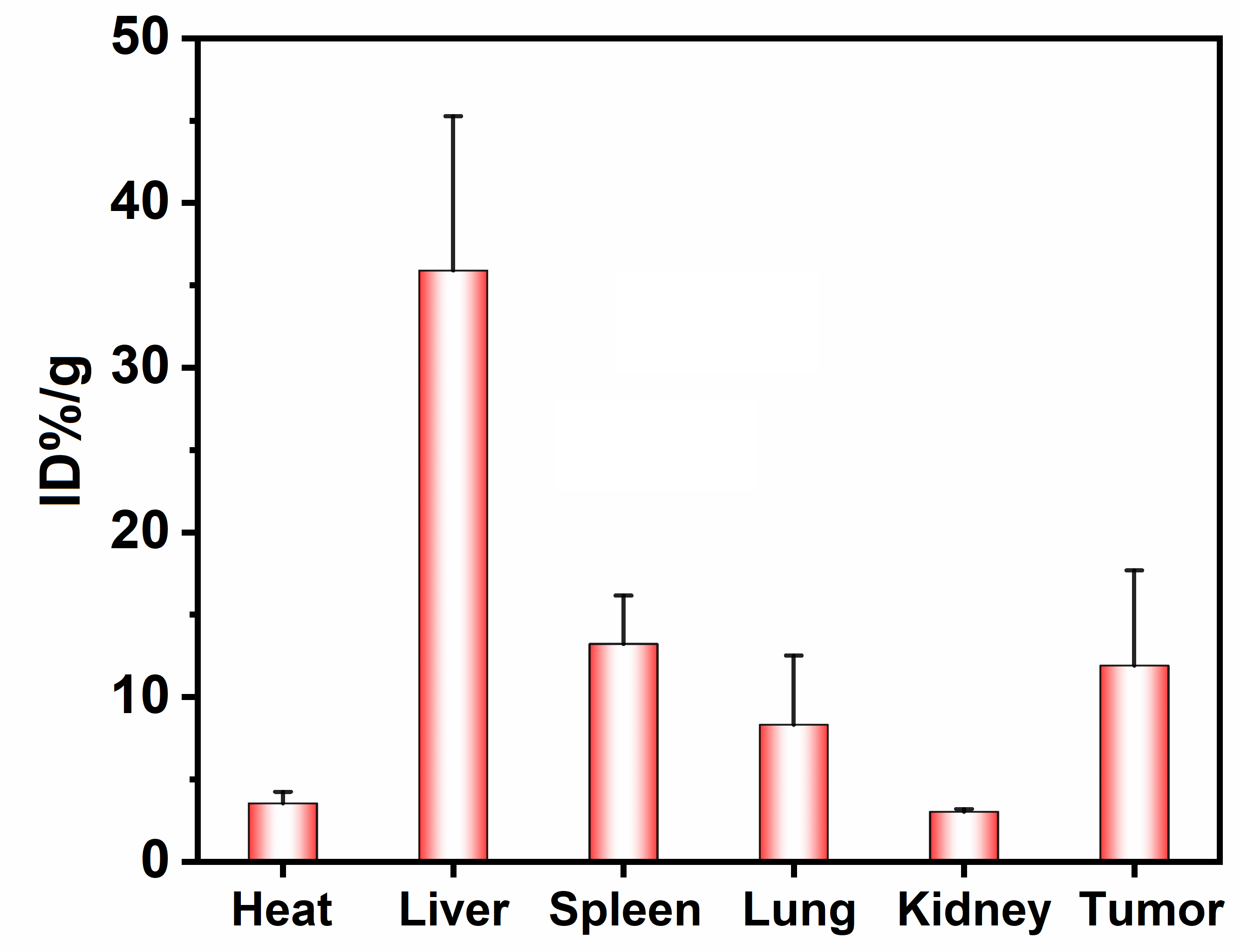
**

**Figure S13.** The Au uptake content in the organs and tumor distribution were evaluated by ICP-MS analysis. After being injected with UGs@PEG (n = 4, dose = 20 mg/kg, mouse body weight) intravenously, tissues were harvested and broken up from mice bearing tumors at 24 h postinjection.


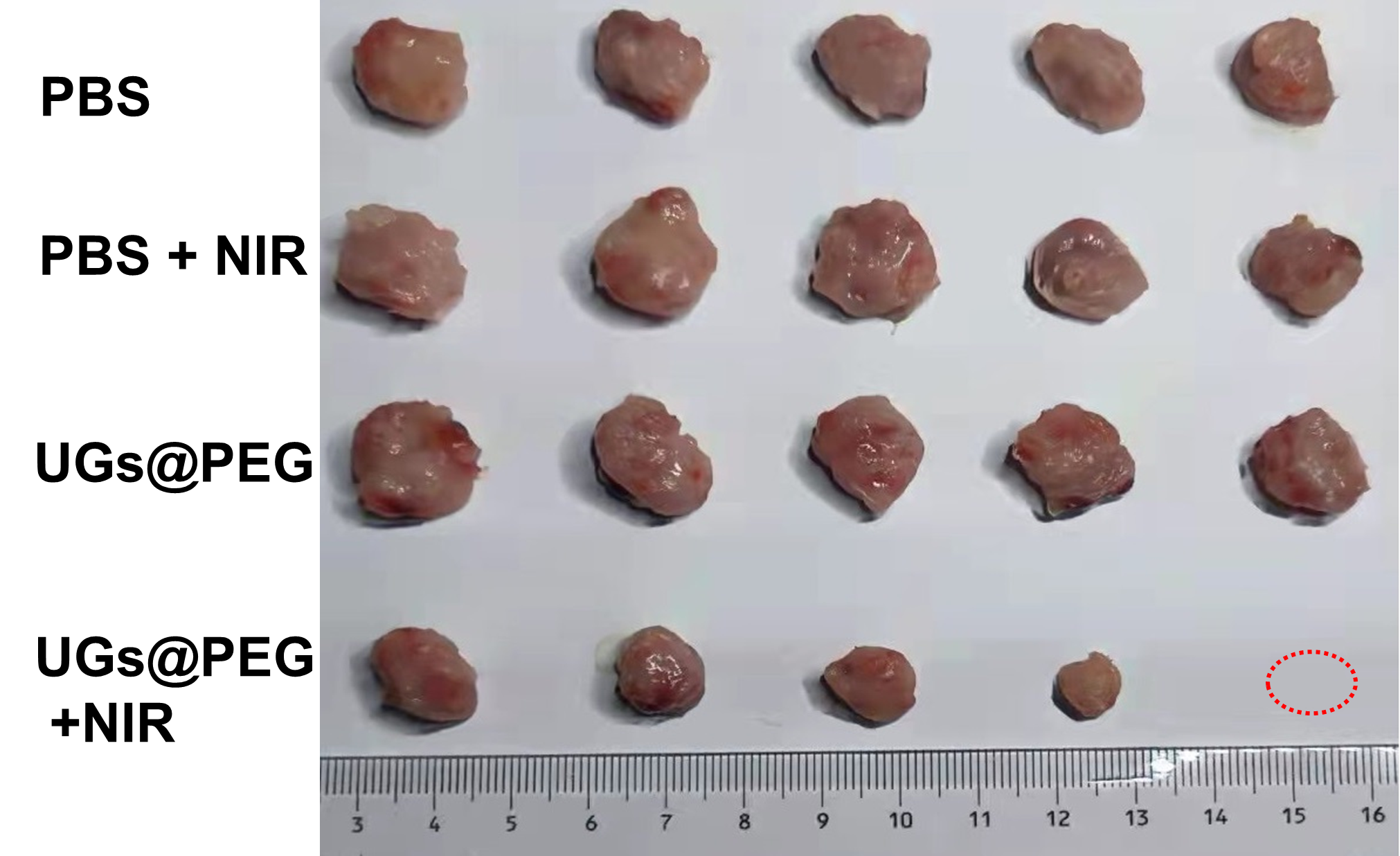


**Figure S1****4** The tumors recorded after the therapeutic process show their varying sizes and thus different therapeutic efficacies.


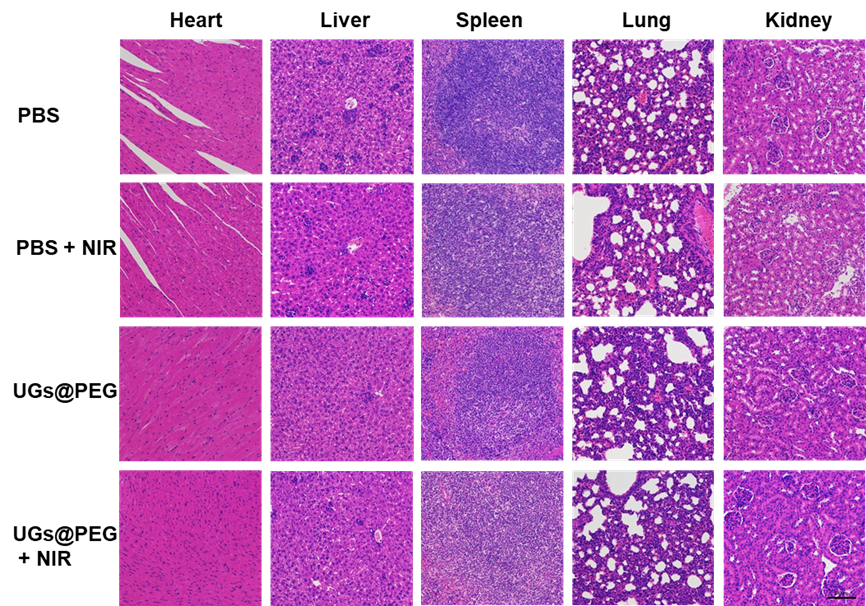


**Figure S15** Hematoxylin and eosin (HE) staining of main organs from all experimental groups: PBS, PBS + NIR, UGs@PEG, and UGs@PEG + NIR. Scale bar: 100 *μ*m.

**Table S1.** UV-Vis absorption peak and photothermal conversion efficiencies of reported gold nanoshells.

| Materials | Description | Photothermal conversion efficiencies, η | UV-Vis absorption peak | Reference |
| --- | --- | --- | --- | --- |
| Silica@Au nanoshells | Core (silica) coated with  a metallic layer (gold) | 8.25% | 800 nm | [S1] |
| mSiO_2_@Au nanoshells | A hollow gold nanoshells | 21.00% | 600-800 nm | [S2] |
| GNShells | Hollow spherical GNShells | 21.77% | 600-800 nm | [S3] |
| PGNSs | Porous gold  nanoshells | 22.43% | 600-800 nm | [S4] |
| Pt-MOF@GNSs | Pt-decorated MOF@gold shells | 38.55% | 800 nm | [S5] |
| Gold nanoshells | A hollow gold nanoshells | 45.00% | 832 nm | [S6] |
| AuNPs-BA-Lips | Gold-nanobranched coated betulinic acid liposomes | 55.70% | 520 nm | [S7] |
| Pt@Au-TP-Lips | Platinum/gold bimetallic-nanoshell-coated triptolide | 56.50% | 650 nm | [S8] |
| PUA | Pt@UiO-66-NH_2_@Au_shell_ nanoparticles | 58.65% | 800-810 nm | [S9] |
| SPIO-HGNS | Iron oxide-enclosed hollow gold nanoshell | 60.00% | 945 nm | [S10] |
| UGs@PEG | **Diffusion growth mechanism of gold nanoshells** | **74.03%** | **Beyond 1300 nm** | **this work** |

**Supplementary References**

[1] F.Y. Cheng, C.T. Chen, C.S. Yeh, Comparative Efficiencies of Photothermal Destruction of Malignant Cells Using Antibody-Coated Silica@Au Nanoshells, Hollow Au/Ag Nanospheres and Au Nanorods. Nanotechnology 20 (2009) 425104.

[2] M. Emamzadeh, G. Pasparakis, Polymer Coated Gold Nanoshells for Combinational Photochemotherapy of Pancreatic Cancer with Gemcitabine. Sci. Rep. 11 (2021) 1−15.

[3] Q. You, Q. Sun, M. Yu, J.P. Wang, S.Y. Wang, L. Liu, Y. Cheng, Y.D Wang, Y.L. Song, F.P. Tan, N. Li, BSA–Bioinspired Gadolinium Hybrid-Functionalized Hollow Gold Nanoshells for NIRF/PA/CT/MR Quadmodal Diagnostic Imaging-Guided Photothermal/Photodynamic Cancer Therapy. ACS Appl. Mater. Interfaces 9 (2017) 40017−40030.

[4] P.C. Xu, R. Wang, W.Q. Yang, Y.Y. Liu, D.S. He, Z.X. Ye, D.Q. Chen, Y. Ding, J.S. Tu, Y. Shen, A DM1-doped Porous Gold Nanoshell System for NIR Accelerated Redox-Responsive Release and Triple Modal Imaging Guided Photothermal Synergistic Chemotherapy. J. Nanobiotechnology 19 (2021) 1−19.

[5] Q. You, K.Y. Zhang, J.Y. Liu, C.L. Liu, H.Y. Wang, M.T. Wang, S.Y. Ye, H.Q. Gao, L.T. Lv, C. Wang, L. Zhu, Y.L. Yang, Persistent Regulation of Tumor Hypoxia Microenvironment via a Bioinspired Pt-Based Oxygen Nanogenerator for Multimodal Imaging-Guided Synergistic Phototherapy. Adv. Sci. 7 (2020) 1903341.

[6] Y.N. Guan, Z. Xue, J.L. Liang, Z.Z. Huang, W.S. Yang, One-pot Synthesis of Size-Tunable Hollow Gold Nanoshells via APTES-in-Water Suspension. Colloids Surf. A Physicochem. Eng. Asp. 502 (2016) 6−12.

[7] Y.P. Liu, X.W. Zhang, L.Y. Luo, L. Li, R.Y. Zhu, A.S. Li, Y.C. He, W.W. Cao, K. Niu, H. Liu, J.Y. Yang, D.W. Gao, Gold-Nanobranched-Shell Based Drug Vehicles with Ultrahigh Photothermal Efficiency for Chemo-Photothermal Therapy. Nanomedicine 18 (2019) 303−314.

[8] L.Y. Luo, H.Y. He, C.H. Li, Y.Q. He, Z.N. Hao, S. Wang, Q.Q. Zhao, Z.W. Liu, D.W. Gao, Near-Infrared Responsive Bimetallic Nanovesicles for Enhanced Synergistic Chemophotothermal Therapy. ACS Biomater. Sci. Eng. 5 (2019) 1321−1331.

[9] C. Liu, L.J. Luo, L.Y. Zeng, J. Xing, Y.Z, Xia, S. Sun, L.Y. Zhang, Z. Yu, J.L. Yao, Z.S. Yu, O.U. Akakuru, M. Saeed, A.G. Wu, Porous Gold Nanoshells on Functional NH_2_-MOFs: Facile Synthesis and Designable Platforms for Cancer Multiple Therapy. Small 14 (2018) 1801851.

[10] M.Z. Yuan, X.H. Feng, T.H. Yan, J.F. Chen, X.Z. Ma, P. Cunha, S.F. Lan, Y. Li, H.C. Zhou, Y. Wang, Superparamagnetic iron oxide-enclosed hollow gold nanostructure with tunable surface plasmon resonances to promote near-infrared photothermal conversion. Adv. Compos. Mater. (2022) 1−12.
